# Copper Integrated PDA-TA Nanocoating via One-Step Rapid Polymerization on Titanium for Anti-Thrombotic and Antibacterial Properties

**DOI:** 10.3390/biom16070953

**Published:** 2026-06-27

**Authors:** Chuangxin Huang, Xin Liu, Zerong Zhang, Yanjun Liu, Qi Chen, Jianli Meng, Qiuliang Wang

**Affiliations:** 1 Ganjiang Innovation Academy, Chinese Academy of Sciences, Ganzhou 341119, China; chxinhuang@mail.ustc.edu.cn (C.H.); zrzhang@gia.cas.cn (Z.Z.); yjliu24@gia.cas.cn (Y.L.); qchen@gia.cas.cn (Q.C.); jlmeng@gia.cas.cn (J.M.); qiuliang@gia.cas.cn (Q.W.); 2 School of Rare Earths, University of Science and Technology of China, Hefei 230026, China; 3 Institute of Electrical Engineering, Chinese Academy of Sciences, Beijing 100190, China

**Keywords:** polydopamine, copper ion, tannic acid, titanium, antibacterial

## Abstract

Long-term clinical translation of left ventricular assist devices (LVADs) is severely hampered by thromboembolism and device-related infection, both originating from inadequate biocompatibility of the device-blood interface. Current titanium surface modifications fail to simultaneously deliver durable antithrombotic and antibacterial performance, while conventional polydopamine-copper (PDA-Cu) coatings suffer from inherent limitations. Herein, we report a one-step rapid co-polymerization strategy based on mussel-inspired polyphenol chemistry to fabricate a copper-integrated polydopamine/tannic acid nanocoating on titanium (Ti/PDT(Cu)). By incorporating tannic acid rich in catechol/pyrogallol moieties, we achieve synergistic acceleration of dopamine oxidative polymerization with copper ions, dramatically shortening the fabrication time to 8 h (vs. 24 h for traditional PDA coatings). This process simultaneously constructs a robust dual-crosslinked network through covalent/hydrogen bonds and metal-phenolic coordination, exhibiting a uniform nanoscale-roughened structure. Comprehensive physicochemical characterizations confirm homogeneous coating deposition, excellent hydrophilicity, uniform Cu distribution, and superior long-term structural stability (95.68% thickness retention after 7 days of physiological immersion). The optimized coating displays broad-spectrum and durable antibacterial activity, with 92.79% and 89.73% reduction of *E. coli* and *S. aureus* at 24 h, respectively, and retains >89% antibacterial efficacy after 7 days of continuous elution (*n* = 3, * *p* < 0.05). Moreover, the coating enables stable and sustained catalytic nitric oxide generation (43.85 ± 2.36 μM cumulative release over 14 days) that mimics endothelial function, resulting in 69.4% inhibition of platelet adhesion and an ultralow hemolysis ratio of 0.97% (*n* = 3). Critically, it maintains excellent cytocompatibility with L929 fibroblasts (>90% cell viability after 72 h co-culture). This work addresses key limitations of conventional PDA-based functional coatings, realizes synergistic antithrombotic and antibacterial dual functions showing great potential for blood-contacting cardiovascular device applications, and provides a facile and robust surface engineering platform for long-term implantable cardiovascular devices.

## 1. Introduction

Heart failure (HF) represents the terminal stage of the cardiovascular disease continuum and has emerged as a formidable global public health challenge [1,2]. In China alone, over 13 million people currently live with HF, and this number is projected to climb to 22.7 million by 2035 [3]. Against this backdrop, left ventricular assist devices (LVADs) have become a first-line clinical strategy for end-stage HF, serving as a bridge to transplantation and recovery, or long-term destination therapy that substantially extends survival and improves quality of life [4,5]. Despite their clinical success, two life-threatening complications—thromboembolism and device-related infection—remain the principal bottlenecks limiting the long-term application of LVADs [6]. Current management, which largely relies on systemic anticoagulation and broad-spectrum antibiotics, can only partially mitigate these risks. It fails to address the underlying etiology and introduces severe adverse events such as intracranial hemorrhage and the emergence of antibiotic-resistant bacteria [7,8]. Converging evidence has established that the fundamental pathological origin of both complications is the inadequate biocompatibility of the device–blood/tissue interface. At the mechanistic level, LVAD-associated thrombosis originates from rapid nonspecific protein adsorption onto the material surface upon blood contact [9,10]. Plasma proteins, particularly fibrinogen and von Willebrand factor (vWF), undergo conformational changes that expose platelet-binding motifs, thereby triggering platelet adhesion, activation, and aggregation. Concurrently, the adsorbed protein layer and activated platelets cooperatively initiate both the intrinsic and extrinsic coagulation cascades, culminating in fibrin network formation and thrombotic occlusion. Consequently, constructing a surface coating that simultaneously integrates durable antithrombotic and broad-spectrum antibacterial functions represents a pivotal route to addressing the clinical challenge of LVADs and improving patient prognosis.

To reduce the risks associated with systemic anticoagulation, surface anticoagulant modification has become a research hotspot. Existing strategies mainly include: Heparinized coatings, the most widely used in clinical practice, but they carry the risk of heparin-induced thrombocytopenia (HIT), and heparin is prone to leaching during long-term implantation, leading to continuous attenuation of anticoagulant activity [11,12]. Zwitterionic/hydrophilic coatings, which inhibit nonspecific protein adsorption through the surface hydration layer, but their long-term stability is poor under the high shear stress environment of LVADs, with a high risk of delamination from the substrate [13,14]. In addition, they lack active anticoagulant activity and cannot counteract high shear stress-induced platelet activation. Endothelialization modification, which mimics the physiological anticoagulant function of native vascular endothelium and represents an ideal anticoagulant strategy [13,15,16]. However, it is limited by long endothelial cell culture cycles, susceptibility to detachment under high shear stress, and difficulty in large-scale clinical translation. In this context, nitric oxide (NO)-mediated anticoagulant strategies have shown tremendous clinical translation potential. NO is a key endogenous anticoagulant molecule continuously secreted by healthy vascular endothelial cells [17]. At physiological concentrations (0.5–4 × 10^−10^ mol/cm^2^·min), it exerts a potent inhibitory effect on platelet adhesion and aggregation via the cGMP signaling pathway [18]. Meanwhile, it possesses multiple biological functions, including inhibition of smooth muscle cell proliferation, anti-inflammation, and promotion of endothelial repair, without increasing bleeding risk, which aligns well with the anticoagulant requirements of LVADs [13,19]. Copper ions (Cu^2+^/Cu^+^) are the natural catalyst for S-nitrosothiols (RSNO) decomposition to generate NO under physiological conditions [20,21]. Through the Cu^2+^/Cu^+^ redox cycle, they can efficiently catalyze NO release from RSNOs under physiological conditions, and their catalytic activity can be precisely regulated by the coordination environment.

Device-related infection is another major life-threatening complication of long-term LVAD implantation, whose pathological core is bacterial adhesion and biofilm formation on the material surface. Existing surface antibacterial modification strategies have critical limitations that fail to meet the requirements of long-term LVAD implantation: antibiotic-loaded coatings show prominent short-term antibacterial efficacy but have short release cycles and high risk of inducing multidrug-resistant bacteria [22,23]; cationic antibacterial agents (e.g., quaternary ammonium compounds, guanidines) kill bacteria via disrupting bacterial membranes with low drug resistance potential yet suffer from poor hemocompatibility, high hemolysis risk and cytotoxicity [24,25]; and the clinically most widely used broad-spectrum silver-based antibacterial coatings pose potential organ accumulation and cytotoxicity risks from sustained silver ion burst release, and more critically, silver ion-induced platelet activation markedly elevates thrombosis risk, which is inherently incompatible with the anticoagulant requirements of LVADs [26,27,28]. In this context, copper ion-mediated antibacterial strategies have shown unique advantages. Copper is an essential trace element for the human body, with a recommended daily intake of 1–2 mg for adults [29]. At physiological concentrations, it exhibits broad-spectrum antibacterial activity against Gram-positive bacteria, Gram-negative bacteria, and MDR strains [30,31,32]. Its antibacterial mechanisms cover multiple dimensions: (1) disrupting bacterial cell membrane integrity and causing intracellular content leakage; (2) interfering with DNA replication and transcription to inhibit bacterial proliferation; (3) blocking energy metabolism by inhibiting key respiratory chain enzymes; (4) inducing reactive oxygen species (ROS) production to damage bacterial biomacromolecules; (5) disrupting mature biofilms and inhibiting new biofilm formation. Most critically, copper ions possess both NO catalytic anticoagulant activity and broad-spectrum antibacterial activity, enabling “single component, dual-function synergy”, which perfectly fits the dual core requirements of anticoagulation and antibacterial for LVADs, and resolves the core contradiction between existing antibacterial strategies and anticoagulant requirements [33,34]. Metal-phenolic networks (MPNs) provide an ideal platform for efficient and stable immobilization of copper ions. MPNs are organic-inorganic hybrid networks self-assembled through the coordination interaction between phenolic hydroxyl groups of polyphenol molecules and metal ions [35]. They have the core advantages of simple preparation process (aqueous phase, ambient temperature and pressure, one-step method), strong substrate universality (can be modified on almost all inorganic and organic substrates), strong adhesion to the substrate, excellent biocompatibility, high metal ion loading capacity, and controllable release kinetics [36,37,38]. They have become a research hotspot in the surface multifunctional modification of medical implantable devices. Among them, polydopamine (PDA), a mussel adhesive protein-mimetic polyphenol polymer formed by oxidative self-polymerization of dopamine in a weakly alkaline environment, is rich in a large number of catechol and amine groups on the surface [39]. It can chelate copper ions through strong coordination interactions, achieving high loading and high stability of copper ion immobilization [40]. Meanwhile, PDA itself has excellent substrate adhesion, biocompatibility, anti-inflammatory and antioxidant activities, making it an ideal carrier for the construction of copper-based dual-function coatings.

However, existing PDA-Cu coatings still have non-negligible drawbacks that limit their application in LVADs [41,42,43]. The oxidative self-polymerization rate of traditional PDA is slow, usually requiring a preparation period of more than 24 h, and the formed coating has a smooth surface and small specific surface area, which limits the copper ion loading and the exposure of catalytic active sites. Subsequently, the coordination mode between copper ions and single PDA is single, and the release kinetics of copper ions are difficult to precisely regulate, which is prone to initial burst release and insufficient late release, making it impossible to balance long-term antibacterial activity and NO catalytic efficiency. Additionally, single PDA coating has poor degradation resistance in physiological blood environment, and is easy to delaminate during long-term implantation, leading to rapid loss of function. Finally, most existing studies only focus on the optimization of single antibacterial or anticoagulant function, failing to achieve the synergistic regulation of dual functions, and have rarely been systematically designed and verified for the special working conditions of high shear stress and long-term implantation of LVADs.

Herein, to address the critical limitations of conventional PDA-Cu coatings and meet the unmet clinical demand for simultaneous long-term antithrombotic and antibacterial dual functions in blood-contacting cardiovascular devices, we developed a facile one-step rapid co-polymerization strategy based on mussel-inspired polyphenol chemistry to construct a copper-integrated polydopamine/tannic acid (Ti/PDT(Cu)) nanocomposite coating on titanium substrates, the most widely used metallic material for LVADs. By incorporating natural plant-derived TA with abundant pyrogallol and catechol moieties, we synergistically accelerated the oxidative self-polymerization of dopamine with copper ions, drastically shortening the fabrication period from more than 24 h for traditional PDA coatings to only 8 h, while constructing a nanoscale roughened surface with high specific surface area to provide sufficient active sites for copper ion immobilization and catalytic activity exposure. Meanwhile, the dual cross-linked network formed by covalent/hydrogen bonding between DA and TA, as well as stable metal coordination chelation between polyphenol groups and copper ions, was designed to realize uniform dispersion and sustained and controlled release of copper ions, while significantly improving the structural stability and degradation resistance of the coating in physiological environments. In this work, we systematically characterized the chemical composition, surface microstructure, physicochemical properties and long-term physiological stability of the as-fabricated coatings, and comprehensively evaluated their in vitro antibacterial performance against clinically relevant Gram-negative *E. coli* and Gram-positive *S. aureus*, nitric oxide catalytic release activity, antithrombotic properties, cytocompatibility and hemocompatibility in strict accordance with ISO 10993 standards [44]. This work aims to address key limitations of traditional PDA-based functional coatings, realize the synergistic regulation of antithrombotic and antibacterial dual functions with potential for LVAD application scenarios, and provide a facile, scalable and robust surface modification platform for long-term implantable cardiovascular medical devices.

## 2. Experimental Section

### 2.1. Materials and Chemicals

Commercially pure Titanium (Ti) foils (99.7 at%, grade 2) with dimensions of 5 mm × 5 mm × 1 mm were purchased from Shanghai Institute of Optics and Fine Mechanics, Chinese Academy of Sciences (Shanghai, China). Dopamine hydrochloride (DA, 98%), Tris(hydroxymethyl) aminomethane hydrochloride (Tris-HCl, ≥99%), S-nitrosoglutathione (GSNO), and L-glutathione (GSH) were obtained from Sigma-Aldrich (Shanghai, China). Copper chloride dihydrate (CuCl_2_·2H_2_O, 99.9% metals basis) was purchased from Shanghai Aladdin Biochemical Technology Co., Ltd. (Shanghai, China). Gram-negative Escherichia coli (*E. coli*, ATCC25922) and Gram-positive Staphylococcus aureus (*S. aureus*, ATCC29213) were provided by Sangon Biotech Co., Ltd. (Shanghai, China). Mouse fibroblast cell line (L929) was purchased from the Cell Bank of Chinese Academy of Sciences (Shanghai, China). Luria–Bertani (LB) broth powder and agar were supplied by Sangon Biotech Co., Ltd. (Shanghai, China). HUVECs (iCell-h110) and the special culture medium for HUVECs (iCell-h110-001b) were purchased from iCell Bioscience Inc. (Shanghai, China). Cell Counting Kit-8 (CCK-8), Live/Dead cytotoxicity kit, and total nitric oxide assay kit were purchased from BestBio Biotechnology Co., Ltd. (Shanghai, China). All other chemicals not mentioned were of analytical grade and commercially available from Sinopharm Chemical Reagent Co., Ltd. (Shanghai, China). Deionized (DI) water (18.2 MΩ·cm) was used throughout the experiment.

### 2.2. Preparation of Ti/PDT(Cu)

#### 2.2.1. Pretreatment of Ti Substrates

Commercially pure Ti foils were first polished sequentially with metallographic sandpapers of 400#, 1000#, and 2000# grit to eliminate surface scratches and achieve a smooth finish. The polished Ti foils were subjected to ultrasonic cleaning in acetone, anhydrous ethanol, and DI water for 15 min each to remove surface oil, organic contaminants, and abrasive residues. After cleaning, the substrates were dried in a vacuum drying oven at 45 °C for 8 h to obtain moisture-free, pretreated Ti samples.

#### 2.2.2. One-Step Co-Deposition of Ti/PDT(Cu) Composite Coatings

First, DA and TA monomers were all dissolved in 50 mL of Tris buffer solution (50 mM, pH 8.5). Then, CuCl_2_·2H_2_O was dosed sequentially with a final concentration of 0.2 mM. The concentration of DA was kept at 2 mg/mL, and the mass ratio of DA/TA was 5:1. Pretreated Ti samples were first pre-wetted with ethanol and then immersed in the above solutions in an air oscillator at room temperature and 120 rpm for 8 h. After that, the samples were washed with DI water and dried at 55 °C for 8 h. In this study, the pure Ti substrate, single DA coating (Ti/PD), a DA coating with copper ions (Ti/PD(Cu)) and a DA/TA coating (Ti/PDT) were also prepared on the Ti surface using the same method as the control.

### 2.3. Surface Characterizations

The polymerization kinetics of the DA/TA/Cu system were monitored by an enhanced double-beam UV-visible (UV-vis) spectrophotometer (TU-1901, Shanghai Spectrum Analyzer Co., Ltd., Shanghai, China), with the absorbance of the reaction solution at 420 nm recorded at preset time intervals within 10 min. The thickness of the composite coatings was measured by spectroscopic ellipsometry (M-2000v, J. A. Woollam Co., Inc., Lincoln, NE, USA) at five random positions on each sample, and the average value was calculated for statistical analysis. The chemical structure of the coatings was characterized by attenuated total reflectance Fourier transform infrared spectroscopy (ATR-FTIR, Nicolet iS10, Thermo Fisher Scientific Inc., Waltham, MA, USA) in the wavenumber range of 4000–500 cm^−1^ with a resolution of 1 cm^−1^. The surface chemical composition and elemental bonding states were analyzed by an X-ray photoelectron spectrometer (XPS, K-Alpha, Thermo Scientific, Waltham, MA, USA) with Al Kα radiation (hν = 1486.6 eV). The survey spectra were recorded over a binding energy range of 0–1200 eV, and high-resolution spectra of C 1s, O 1s, N 1s, and Cu 2p were acquired for detailed peak fitting analysis. The generation of free radicals during DA polymerization was detected by electron paramagnetic resonance spectroscopy (EPR, Bruker Elexsys E500, Bruker BioSpin GmbH, Rheinstetten, German) at room temperature, with the test parameters set as: center field 3510 G, sweep width 100 G, microwave frequency 9.85 GHz, and modulation amplitude 1 G. The surface morphology of the samples was observed using a field emission scanning electron microscope (FESEM, JSM-IT800, JEOL Ltd., Akishima, Tokyo, Japan) with an accelerating voltage of 10 kV. Prior to SEM observation, all samples were sputter-coated with a 10 nm gold layer to improve electrical conductivity. The elemental distribution of the coatings was characterized by energy-dispersive X-ray spectroscopy (EDS) equipped on the FESEM, with elemental mapping performed at a magnification of 10,000×. The three-dimensional surface topography and roughness parameters (root-mean-square roughness, Rq; average roughness, Ra) were determined by an atomic force microscope (AFM, Dimension Icon, Bruker, Germany) in tapping mode with a silicon nitride probe (RTESPA-300, k = 40 N/m). The surface hydrophilicity of the coatings was evaluated using a contact angle system (DSA HT1600, KRÜSS GmbH, Hamburg, Germany) at 25 °C. A 2 μL droplet of ultrapure water was deposited on the sample surface, and the contact angle was recorded after 3 s of stabilization; each sample was measured at three random positions, and the average value was reported. Three different areas (5 μm × 5 μm) on each sample were scanned, and the average roughness values were calculated. The surface zeta potential of the samples was measured using a zeta potential analyzer (Zetasizer Nano ZS90, Malvern Panalytical Ltd., Malvern, UK) in 10 mM KCl solution at pH 7.4, with three parallel measurements for each sample. To evaluate the long-term structural stability of the coatings, Ti/PDT(Cu) samples (optimized group) were immersed in 10 mL of phosphate-buffered saline (PBS, pH 7.2) and oscillated at 120 rpm at 37 °C for 7 days. The coating thickness was measured at 1–7 days to monitor the changes in structural stability.

### 2.4. Antibacterial Activity

#### 2.4.1. Bacterial Culture Preparation

The antibacterial activity was evaluated using the colony-forming unit (CFU) counting method for antibacterial activity assessment on plastics and non-porous surfaces. Gram-negative *E. coli* and Gram-positive *S. aureus* were used as model bacteria to evaluate the antibacterial activity of the coatings. The bacteria were first inoculated into LB broth and incubated at 37 °C with shaking at 150 rpm for 12 h to obtain a logarithmic-phase bacterial suspension. The bacterial concentration was adjusted to 1.0 × 10^6^ CFU/mL using sterile PBS solution (pH 7.4) based on the absorbance measurement at 600 nm (OD_600_ ≈ 0.1).

#### 2.4.2. Antibacterial Assay (Colony-Forming Unit)

The antibacterial activity was evaluated using the CFU counting method. All samples (pure Ti, Ti/PD, Ti/PDT, Ti/PD(Cu), Ti/PDT(Cu)) were sterilized by ultraviolet radiation (254 nm) for 30 min prior to the assay. Each sterilized sample was placed in a 24-well plate, and 400 μL of the adjusted bacterial suspension (1.0 × 10^6^ CFU/mL) was added to each well, ensuring full contact between the bacterial suspension and the sample surface. The 24-well plate was incubated at 37 °C for 24 h under static conditions. After incubation, the samples were gently rinsed with sterile PBS three times to remove non-adherent bacteria. Then, each sample was transferred to a centrifuge tube containing 4 mL of sterile PBS, and the adherent bacteria were detached by vortexing at 2000 rpm for 5 min. The bacterial suspension was serially diluted 10-fold with sterile PBS, and 100 μL of the diluted suspension (10^−3^, 10^−4^, 10^−5^ dilutions) was spread evenly on LB agar plates. The agar plates were incubated at 37 °C for 18–24 h, and the number of visible bacterial colonies was counted. To evaluate the long-term antibacterial stability of the coatings, all samples were immersed in 10 mL of phosphate-buffered saline (PBS, pH 7.2) and oscillated at 120 rpm at 37 °C for 7 days for antibacterial assay. The antibacterial rate was calculated using the following formula:

$$Antibacterial\;rate\left(\%\right)=\left(1-\frac{CFU\;of\;modified\;sample}{CFU\;of\;pristine\;substrate}\right)\times 100\%$$

Each experiment was performed in triplicate, and the results were expressed as mean ± standard deviation (SD).

### 2.5. Cytotoxicity Assay

According to the previously reported literature, the cytocompatibility of the engineered samples with L929 mouse skin fibroblasts was systematically evaluated using CCK-8 proliferation assays and Live/Dead viability staining. Following high-pressure steam sterilization (121 °C, 30 min), samples were aseptically transferred to 24-well plates. L929 cells in logarithmic growth phase were harvested and seeded at optimized densities of 4 × 10^4^ cells/well in complete Dulbecco’s modified Eagle’s medium (DMEM) supplemented with 10% fetal bovine serum (FBS) and 1% penicillin-streptomycin, with triplicate wells established per experimental group to ensure statistical reliability. After 2 h co-incubation at 37 °C/5% CO_2_ to ensure cellular attachment, sample-loaded groups were supplemented with fresh culture medium, and followed by co-cultured with the cells for 24 h and 72 h test intervals under standardized culture conditions (37 °C, 5% CO_2_, 95% humidity). Then, for CCK-8 quantification, the metabolic assay was initiated by aspirating spent medium, followed by three gentle PBS washes to remove non-adherent cells. A 10% CCK-8 solution in phenol red-free DMEM (1 mL/well) was introduced and incubated for 2 h under culture conditions. Subsequently, 100 μL aliquots of supernatant from each well were transferred to 96-well plates for optical density measurement at 450 nm using a microplate reader (Multiskan FC, Thermo Scientific, USA), with background subtraction at 600 nm. Parallel Live/dead assessments employed Calcein-AM/PI dual staining according to manufacturer protocols. Briefly, the culture medium was removed, and the cells were rinsed with PBS once. Then, 1 mL of staining solution (Calcein-AM:PI = 1:1000 in PBS) was added to each well, and the cells were incubated at 37 °C for 30 min in the dark. Viability profiles were then quantified via fluorescence imaging using an FV1200 inverted microscope (Olympus Corporation, Shinjuku, Tokyo, Japan), with live cells emitting green fluorescence (ex/em 490/515 nm) and dead cells exhibiting red fluorescence (ex/em 535/617 nm). Image analysis was performed using ImageJ software 1.54u2 with cell counting plugins to determine viability percentages. The experiment was repeated three times for statistical analysis.

### 2.6. Measurement of Catalytic NO Release

The NO release behavior catalyzed by the Cu^2+^-immobilized coatings was analyzed using the Griess reagent method with a total nitric oxide assay kit. Before the experiment, all samples were immersed in 1% SDS for 30 min, rinsed thoroughly with DI water for 5 times, and then balanced with sterile PBS three times. Briefly, the pristine and modified Ti samples were incubated in 1 mL of PBS solution containing 10 μM GSNO (NO donor) and 10 μM GSH (reducing agent) at 37 °C in the dark. At preset time points (1–14 day), 50 μL of the incubation solution was collected, and an equal volume of fresh PBS containing GSNO and GSH was replenished. For the Griess assay, the collected sample solution was added to a 96-well plate, followed by the sequential addition of 50 μL of diluted Griess Reagent I and Griess Reagent II. After 15 min of incubation at room temperature in the dark, the absorbance of the mixed solution at 540 nm was measured using a microplate reader (Multiskan FC, Thermo Scientific, USA). The cumulative concentration of NO was calculated from a calibration curve constructed with known concentrations of NaNO_2_, and the experiment was performed in triplicate.

### 2.7. Hemolysis Test

Fresh rabbit blood was obtained from Nanjing Senberga Biotechnology Co., LTD. The hemocompatibility of the pristine and modified Ti surfaces was evaluated via a hemolysis test using red blood cells (RBCs) collected from fresh rabbit blood, in accordance with ISO 10993-4 guidelines [45]. Fresh rabbit blood was collected into a sodium citrate anticoagulant tube and centrifuged at 3000 rpm for 10 min to separate RBCs. The collected RBCs were diluted 10-fold with sterile normal saline for subsequent experiments. All samples were sterilized by UV radiation for 30 min and placed in 10 mL centrifuge tubes, with 5 mL of normal saline added to each tube and pre-incubated at 37 °C for 30 min. Then, 50 μL of the diluted RBC suspension was added to each sample tube, followed by gentle mixing and incubation at 37 °C for 2 h. Deionized water (5 mL + 50 μL RBC suspension) was used as the positive control, and normal saline (5 mL + 50 μL RBC suspension) was used as the negative control, with three parallel samples set for each group. After incubation, all tubes were centrifuged at 3000 rpm for 5 min, and 200 μL of the supernatant was transferred to a 96-well plate. The absorbance of the supernatant at 545 nm was measured using a microplate reader (Multiskan FC, Thermo Scientific, USA). The hemolysis ratio was calculated using the following formula:

$$Hemolysis\;ratio\left(\%\right)=\frac{{OD}_{sample}-{OD}_{negative}}{{OD}_{positive}-{OD}_{negative}}\times 100\%$$

where ${OD}_{sample}$, ${OD}_{negative}$, and ${OD}_{positive}$ are the absorbance values of the sample group, negative control group, and positive control group at 545 nm, respectively.

### 2.8. Antiplatelet Assessment

Fresh rabbit whole blood was collected into a sodium citrate anticoagulant tube and centrifuged at 1000 rpm for 10 min to obtain platelet-rich plasma (PRP). The platelet-rich plasma (PRP) portion was pipetted out without disturbing the buffy coat layer of the leukocytes. The remaining blood was spun again at 3082 RCF for 20 min to collect platelet-poor plasma (PPP). The total platelet count of the PRP was determined using a HESKA Element-HT5 Hematology Analyzer (Loveland, CO, USA). All samples were sterilized by UV radiation (254 nm) for 15 min and pre-balanced in 5 mL of PBS (pH 7.4) for 12 h. Then, 200 μL of PRP supplemented with 10 μM GSNO and 10 μM GSH was added to the surface of each sample, followed by incubation at 37 °C for 120 min. Following the incubation period, the samples were removed and rinsed with PBS. The surface-adhered platelets were then lysed with a 2% *v*/*v* Triton X-100 solution for 30 min. Afterward, the degree of platelet adhesion was quantified by lactate dehydrogenase released during lysis using a Roche Cytotoxicity Detection Kit (λ = 492 nm with λ_ref_ = 620 nm). A calibration curve was constructed using known dilutions of the final platelet solution. The number of platelets adhered to the sample surface was interpolated from the calibration curve.

### 2.9. Statistical Analysis

In this study, we conducted parallel experiments to measure the thickness of the coatings, surface roughness, water contact angle, zeta potential, antibacterial rate, cell viability, hemolysis ratio, catalytic NO release, and antiplatelet adhesion. A minimum of three parallel specimens were measured simultaneously for each experimental group, and the average value was calculated. The sample size (*n*) for each experiment is provided in the respective figure legend. The assay results were exhibited as mean ± standard deviation (SD) and all error bars in the figures represent SD. Statistical analysis was conducted using SPSS 22.0 software (IBM, USA) with the one-way analysis of variance (ANOVA) method followed by Tukey’s post hoc test. A difference of * *p* < 0.05 was considered statistically significant.

## 3. Results and Discussions

### 3.1. Fabrication and Characterization of the Pure/Modified Ti Surfaces

Surface engineering of Ti substrates with bioactive polydopamine-based coatings has been widely recognized as a promising strategy to mitigate the two most life-threatening clinical complications, namely thromboembolism and implant-associated infection, for left ventricular assist devices (artificial heart pumps) [33,46]. However, the inherently slow oxidative self-polymerization rate of dopamine monomers severely restricts the fabrication efficiency, controllable thickness regulation, and functional loading capacity of pristine polydopamine coatings in practical clinical applications. In this study, tannic acid, a natural plant-derived polyphenol with abundant pyrogallol and catechol moieties, was introduced to accelerate DA polymerization and construct a high-loading functional coating matrix, with the polymerization kinetics and coating formation efficiency systematically monitored via UV-vis spectrophotometry and thickness characterization. The characteristic absorbance at 420 nm, which corresponds to the π-π* transition of the conjugated indole structure formed during DA oxidative cyclization and polymerization, was recorded to quantify the polymerization rate of different reaction systems and the results are shown in Figure S1. Within 40 min of reaction, the pristine DA system exhibited an extremely slow increase in absorbance (only 0.09 at 40 min), indicating the low intrinsic polymerization efficiency of DA monomers under neutral conditions. In comparison, the introduction of TA significantly accelerated the polymerization process, with the absorbance of the DA + TA system reaching 0.65 at 40 min, which was more than 8 times that of the pristine DA system. While Cu^2+^ incorporation also showed a certain promotion effect on DA polymerization (DA + Cu system, absorbance 0.43 at 40 min), the ternary DA + TA + Cu system exhibited the fastest polymerization rate, with the absorbance reaching 0.92 at 40 min, demonstrating the synergistic acceleration effect of TA and Cu^2+^ on DA oxidative polymerization. Consistent with the polymerization kinetics results, the coating thickness after 8 h of deposition (as shown in Figure S2) further verified the significant improvement in film-forming efficiency induced by TA incorporation. The thickness of the pristine Ti/PD coating was only 13.5 nm, while the Ti/PDT coating achieved a thickness of 35.4 nm under the same deposition time. Meanwhile, the Ti/PDT(Cu) coating exhibited the maximum thickness of 42.9 nm, which was more than 3 times that of the Ti/PD coating with a significant difference (* *p* < 0.05). This enhanced film-forming efficiency not only confirms the critical role of TA in regulating DA polymerization but also provides a thicker, more robust coating matrix with abundant active sites for subsequent functional metal ion immobilization. To further elucidate the chemical composition, intermolecular interactions and structural evolution of the as-fabricated functional coatings, Fourier transform infrared (FTIR) spectroscopy, X-ray photoelectron spectroscopy (XPS) and electron paramagnetic resonance (EPR) were systematically performed.

#### 3.1.1. Chemical Structures and Compositions

**Figure 1:**
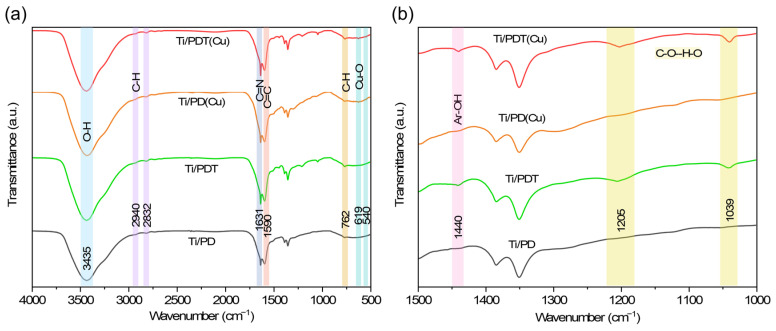
FTIR spectra of functional coating-modified Ti substrates. (**a**) Full-scan spectra recorded in the wavenumber range of 500–4000 cm^−1^ for Ti/PD, Ti/PDT, Ti/PD(Cu), and Ti/PDT(Cu) samples. (**b**) Magnified spectra of the fingerprint region in the wavenumber range of 1000–1500 cm^−1^, showing the characteristic absorption peaks of oxygen-containing functional groups in the polyphenol-based coatings.

FTIR spectroscopy was performed to elucidate the chemical composition, verify the successful stepwise fabrication of polydopamine-based functional coatings, and unravel the intermolecular interactions among DA, TA, and copper ions on Ti substrates. The results of full-scan spectra (4000–500 cm^−1^) and magnified fingerprint-region spectra (1500–1000 cm^−1^) are presented in Figure 1a and 1b, respectively. For the pristine polydopamine-coated Ti sample (Ti/PD), the well-resolved characteristic absorption peaks of polymerized DA were clearly identified, confirming the successful oxidative self-polymerization of DA on the Ti surface. The broad and intense absorption band centered at 3435 cm^−1^ was ascribed to the stretching vibration of O-H bonds, originating from abundant phenolic and alcoholic hydroxyl groups in the polydopamine matrix, as well as physically adsorbed water molecules on the coating surface. The two weak peaks at 2940 cm^−1^ and 2832 cm^−1^ corresponded to the asymmetric and symmetric stretching vibrations of aliphatic C-H bonds in methylene (-CH_2_-) groups of the polydopamine skeleton, respectively. Notably, the distinct peak at 1631 cm^−1^ was attributed to the stretching vibration of C=N bonds derived from the Schiff base structure formed during DA oxidation and cyclization, which is the hallmark of successful polydopamine polymerization. Meanwhile, the peak at 1590 cm^−1^ was assigned to the skeletal stretching vibration of conjugated C=C bonds in the aromatic indole and catechol rings of polydopamine, coupled with the in-plane bending vibration of N-H bonds from primary and secondary amine groups in the polymer matrix. The absorption peak at 762 cm^−1^, corresponding to the out-of-plane bending vibration of aromatic C-H bonds, further validated the retention of the aromatic catechol structure in the as-formed polydopamine coating. Upon the incorporation of TA into the polydopamine matrix (Ti/PDT sample), distinct evolutions of the characteristic peaks were observed, demonstrating the successful co-deposition of TA and DA and the strong intermolecular interactions between the two polyphenol components. Firstly, the O-H stretching band at 3435 cm^−1^ exhibited a significantly enhanced absorption intensity (deeper transmittance valley) in the Ti/PDT sample compared with Ti/PD, which was attributed to the large number of additional phenolic hydroxyl groups introduced by TA—a natural polyphenol with abundant pyrogallol and catechol moieties. This result was further supported by the magnified fingerprint region spectra (Figure 1b), where the absorption peaks at 1440 cm^−1^ (in-plane bending vibration of O-H bonds in aromatic phenolic hydroxyl groups, Ar-OH), 1205 cm^−1^ (stretching vibration of C-O bonds in phenolic hydroxyl groups), and 1039 cm^−1^ (stretching vibration of C-O bonds in aliphatic alcoholic hydroxyl groups and ether linkages) all showed markedly increased intensity in Ti/PDT relative to Ti/PD [45,46]. It is worth noting that the characteristic peaks of the polydopamine matrix (including C=N at 1631 cm^−1^, aromatic C=C at 1590 cm^−1^, and aliphatic C-H at 2940/2832 cm^−1^) were well retained in the Ti/PDT sample, confirming that the introduction of TA did not disrupt the fundamental cross-linked structure of polydopamine, but rather introduced a large number of active hydroxyl sites for subsequent metal ion coordination.

For the Cu-loaded samples (Ti/PD(Cu) and Ti/PDT(Cu)), FTIR results provided direct evidence for the successful immobilization of Cu^2+^ ions via coordination bonding with the polyphenol matrix, as well as the structural stability of the coating after metal ion loading. The most prominent feature was the appearance of two new, distinct absorption peaks at 619 cm^−1^ and 540 cm^−1^ exclusively in the Cu-containing samples, which were assigned to the stretching vibration of Cu-O bonds formed between Cu^2+^ ions and the deprotonated hydroxyl oxygen atoms of the catechol and pyrogallol moieties in polydopamine and TA [43]. This result unambiguously confirmed that Cu^2+^ ions were not simply physically adsorbed on the coating surface, but formed stable chemical coordination bonds with the polyphenol matrix, which is critical for the sustained and controlled release of Cu ions, thus ensuring long-term antibacterial and antithrombotic performance of the functional coatings. In addition to the appearance of Cu-O characteristic peaks, obvious changes in the hydroxyl-related absorption peaks were observed after Cu^2+^ loading. Specifically, the intensity of the O-H stretching band at 3435 cm^−1^, as well as the Ar-OH related peaks at 1440 cm^−1^ and 1205 cm^−1^, decreased slightly in Ti/PD(Cu) and Ti/PDT(Cu) compared with their Cu-free counterparts. This phenomenon was attributed to the deprotonation of phenolic hydroxyl groups during the coordination process, where the oxygen atoms of the hydroxyl groups acted as electron donors to form chelate structures with Cu^2+^ ions, thus reducing the number of free O-H bonds in the coating matrix. Furthermore, slight shifts in the C=N (1631 cm^−1^) and aromatic C=C (1590 cm^−1^) peaks were detected in the Cu-loaded samples, indicating that the amine groups and Schiff base structures in the polydopamine matrix also participated in the coordination with Cu^2+^ ions, further enhancing the binding stability of Cu ions in the coatings [47]. Collectively, the FTIR characterization results systematically validated the successful stepwise fabrication of the four designed functional coatings on Ti substrates.

**Figure 2:**
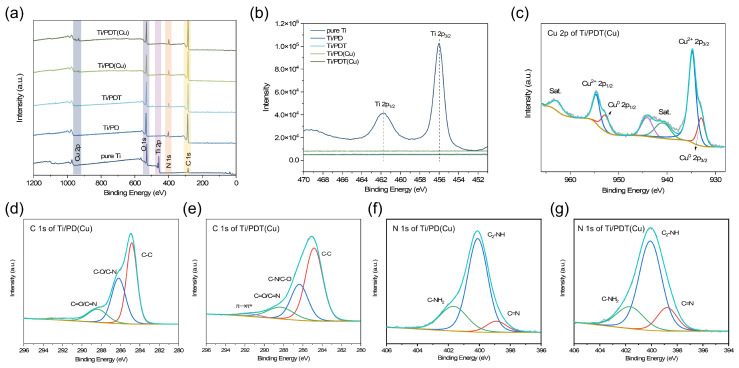
XPS characterization of pure Ti and functional coating-modified Ti substrates. (**a**) Full survey spectra of pure Ti, Ti/PD, Ti/PDT, Ti/PD(Cu), and Ti/PDT(Cu). (**b**) High-resolution Ti 2p spectra of all samples. (**c**) High-resolution Cu 2p spectrum of the Ti/PDT(Cu) coating. (**d**,**e**) High-resolution C 1s spectra of Ti/PD(Cu) and Ti/PDT(Cu). (**f**,**g**) High-resolution N 1s spectra of Ti/PD(Cu) and Ti/PDT(Cu).

To further elucidate the elemental composition, chemical bonding states, and crosslinking mechanism of the as-prepared coatings at the atomic scale, X-ray photoelectron spectroscopy was performed, with the results presented in Figure 2. The full survey spectra (Figure 2a) showed that the pristine pure Ti substrate exhibited characteristic peaks of Ti 2p and O 1s, while all polydopamine-coated samples (Ti/PD, Ti/PDT, Ti/PD(Cu), Ti/PDT(Cu)) displayed dominant C 1s and N 1s peaks, the signature elements of polydopamine, confirming the successful deposition of PDA-based coatings on the titanium surface. Correspondingly, the high-resolution Ti 2p spectra (Figure 2b) showed distinct Ti 2p_3/2_ (~458.5 eV) and Ti 2p_1/2_ (~464.2 eV) peaks for pure Ti, while these peaks were nearly undetectable for all coated samples, indicating that the PDA-based coatings formed uniform, complete coverage on the Ti substrate, consistent with the spectroscopic ellipsometry thickness measurement results. Then, for Cu-incorporated coatings (Ti/PD(Cu) and Ti/PDT(Cu)), distinct Cu 2p characteristic peaks appeared in the survey spectra at 930–960 eV, verifying the successful incorporation of Cu into the PDA matrix. The high-resolution Cu 2p spectrum of Ti/PD(Cu) (Figure S3) and Ti/PDT(Cu) (Figure 2c) was deconvoluted into four main peaks: Cu^2+^ 2p_3/2_ at ~934.5 eV, Cu^0^ 2p_3/2_ at ~932.6 eV, Cu^2+^ 2p_1/2_ at ~954.3 eV, and Cu^0^ 2p_1/2_ at ~952.4 eV, accompanied by characteristic satellite peaks of Cu^2+^ at ~942.5 eV and ~962.1 eV. These results demonstrated that Cu in the coating mainly existed in the divalent state (Cu^2+^), with a small amount of reduced Cu^0^ attributed to the mild reducibility of catechol groups in PDA and TA. The dominant Cu^2+^ species were chelated with catechol and amino groups from DA and TA to form stable metal coordination bonds, which not only enhanced the crosslinking density of the coating to improve structural stability, but also provided a sustained source of antibacterial active Cu^2+^ ions.

The high-resolution C 1s spectra of Ti/PD(Cu) and Ti/PDT(Cu) (Figure 2d,e) were deconvoluted into three main peaks for all PDA-based coatings: C-C/C-H at ~284.8 eV, C-O/C-N at ~286.1 eV, and C=O/C=N at ~288.4 eV. Notably, an additional π→π* transition shake-up satellite peak at ~291.3 eV was observed for Ti/PDT(Cu), a characteristic feature of the aromatic ring structure in TA, directly confirming a significant positive inductive effect of TA and the accelerated structural rearrangement. The co-deposition of TA with abundant catechol groups also accelerated the oxidative polymerization of DA, consistent with the polymerization kinetics results from UV-vis spectroscopy. Moreover, the relative content of C-O/C-N species in Ti/PDT(Cu) was higher than that in Ti/PD(Cu), as TA provides abundant phenolic hydroxyl groups that increase the density of hydrophilic oxygen-containing functional groups on the coating surface, well consistent with the enhanced hydrophilicity observed in the water contact angle test. The high-resolution N 1s spectra (Figure 2f,g) were fitted into three characteristic peaks corresponding to primary amine (C-NH_2_, ~401.8 eV), secondary amine (C_2_-NH, ~400.2 eV), and imine (C=N, ~398.8 eV), the typical nitrogen-containing structures formed during DA oxidative polymerization. Compared with Ti/PD(Cu) and other control groups, Ti/PDT(Cu) exhibited a significantly reduced relative content of C-NH_2_ and an increased proportion of C=N species. This phenomenon is attributed to two synergistic driving factors [48]: (1) The accelerated and more complete oxidative polymerization of DA mediated by TA and Cu^2+^. During DA polymerization, free primary amine groups in DA monomers are continuously consumed via intramolecular cyclization, Schiff base reaction between amine groups and oxidized quinone structures, and subsequent oxidative rearrangement to form conjugated indole structures, which directly convert C-NH_2_ into C_2_-NH and unsaturated C=N species. Cu^2+^ acts as an efficient oxidant to accelerate DA oxidation, while TA promotes quinone-phenol coupling and radical transfer during polymerization, collectively advancing the polymerization process and promoting the conversion of primary amines to imine structures. (2) The chelation interaction between Cu^2+^ and free C-NH_2_ groups further reduces the relative content of free primary amines, while the coordination effect stabilizes polymerization intermediates and facilitates oxidative rearrangement to form C=N-containing conjugated structures. Additionally, as shown in Figure S4, EPR characterization further revealed that the Ti/PDT(Cu) coating exhibited a strong characteristic semiquinone radical signal (g ≈ 2.004), while no obvious radical signal was observed for the Cu-free Ti/PDT coating; this stable radical species is consistent with the enhanced conjugated structure identified by XPS. In summary, XPS and EPR results directly verified the successful co-deposition of TA and Cu into the PDA matrix, and confirmed the construction of a dual crosslinking network consisting of covalent/hydrogen bonding between DA and TA, as well as metal coordination chelation between catechol/amino groups and Cu^2+^. The well-defined chemical composition and crosslinking structure of the coatings provide an atomic-level basis for the subsequent analysis of surface morphology, physicochemical properties, and biological functions.

#### 3.1.2. Surface Morphology Characterization

**Figure 3:**
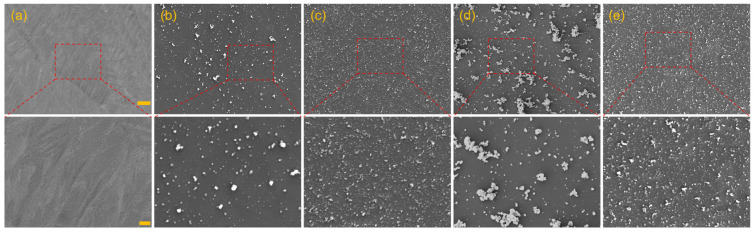
SEM characterization of pure Ti and functional coating-modified Ti substrates. All samples include pure titanium (pure Ti), single polydopamine coating (Ti/PD), polydopamine/tannic acid coating (Ti/PDT), polydopamine coating with copper ions (Ti/PD(Cu)), and copper-integrated polydopamine/tannic acid coating (Ti/PDT(Cu)). (**a**) Pure Ti substrate; (**b**) Ti/PD sample; (**c**) Ti/PDT sample; (**d**) Ti/PD(Cu) sample; (**e**) Ti/PDT(Cu) sample. The upper row shows low-magnification images, and the lower row shows the corresponding high-magnification images of the red dashed box area in the upper row. Scale bar: 4 μm and 1 μm.

SEM was performed to characterize the surface morphology, coating deposition behavior, and microstructure evolution of the as-prepared samples, with the aim of verifying the successful fabrication of functional coatings and revealing the regulatory effects of TA incorporation and copper ion coordination on the coating microstructure. The low and corresponding high-magnification SEM images of pure Ti, Ti/PD, Ti/PDT, Ti/PD(Cu), and Ti/PDT(Cu) samples are systematically presented in Figure 3. The mechanically polished pure Ti substrate (Figure 3a) exhibited an overall flat and smooth surface, with only faint linear scratches originating from the polishing process observed under both low and high magnification. This defect-free, low-roughness surface served as a blank baseline for evaluating the morphological changes induced by the subsequent polyphenol-based coating modification. For the Ti/PD sample (Figure 3b), the surface was fully covered by a continuous polydopamine coating, with the original polishing scratches on the Ti substrate completely masked, confirming the successful oxidative self-polymerization and stable deposition of dopamine on the Ti surface. Under high magnification, the coating was composed of discrete, near-spherical polydopamine nanoparticles with a size range of tens to hundreds of nanometers. These nanoparticles were sparsely and relatively uniformly distributed on the surface, resulting in only a slight increase in surface roughness compared with the pure Ti substrate. This morphological feature is consistent with the classic polymerization behavior of dopamine, where dopamine monomers undergo oxidation, cyclization, and self-assembly to form dispersed nanoparticle aggregates on the material surface, which is also in good agreement with the successful formation of polydopamine verified by our previous FTIR spectroscopy results. Then, incorporation of TA into the polydopamine matrix (Ti/PDT sample, Figure 3c) induced a significant and regular change in the coating microstructure. At low magnification, the coating maintained a continuous, flat, and defect-free appearance without cracks, pinholes, or peeling. Under high magnification, a marked increase in the density and distribution uniformity of polydopamine-derived nanoparticles was observed, compared with the Ti/PD sample. The nanoparticles were more densely and homogeneously packed across the entire surface, forming a uniform nanoscale roughened structure without obvious particle agglomeration. This phenomenon can be attributed to the multiple hydrogen-bonding interactions between TA and dopamine/polydopamine segments, as reported in the previous study on polyphenol chemistry-mediated polydopamine coating modification [49]. As a natural polyphenol with abundant pyrogallol and catechol moieties, TA acts as a cross-linker and growth regulator during dopamine polymerization: it not only provides additional active sites for polydopamine nucleation and growth, but also inhibits the excessive random aggregation of nanoparticles via steric hindrance and hydrogen-bonding stabilization, ultimately leading to a denser, more uniform coating with higher surface active site density. This result is also highly consistent with our previous FTIR findings, where the enhanced hydroxyl-related absorption peaks confirmed the successful introduction of TA and the increased number of active functional groups on the coating surface.

For the Cu-loaded Ti/PD(Cu) sample (Figure 3d), a distinct change in surface morphology was observed after Cu^2+^ coordination. At high magnification, the originally dispersed polydopamine nanoparticles in the Ti/PD coating underwent significant cross-linking and aggregation, forming a large number of irregular, micron-scale particle agglomerates. These agglomerates were heterogeneously distributed on the coating surface, with obvious particle stacking in local areas, while the underlying coating was still visible in other regions. This morphological change is directly related to the coordination interaction between Cu^2+^ and the polydopamine matrix: Cu^2+^ can form multidentate coordination bonds with the catechol hydroxyl groups and amino groups in polydopamine, which cross-links adjacent polydopamine nanoparticles and induce particle aggregation. However, the limited number of active coordination sites in the single polydopamine matrix cannot achieve uniform dispersion of Cu^2+^, thus leading to local excessive cross-linking and heterogeneous agglomeration of the coating microstructure. Notably, the Ti/PDT(Cu) sample (Figure 3e) exhibited a significantly optimized and uniform microstructure after Cu^2+^ loading, which effectively overcomes the agglomeration problem observed in the Ti/PD(Cu) sample. At low magnification, the coating remained continuous and intact, with no obvious structural defects. Under high magnification, the coating was composed of high-density, uniformly sized nanoparticles without significant micron-scale agglomeration, and the overall morphology was highly consistent with that of the Ti/PDT sample, while maintaining a well-defined nanoscale roughened structure. This improvement can be ascribed to the abundant phenolic hydroxyl groups introduced by TA, which provide a large number of additional and uniform coordination sites for Cu^2+^. The stable multi-component coordination structure formed between TA, polydopamine, and Cu^2+^ not only achieves uniform immobilization of Cu^2+^ in the coating matrix, but also effectively inhibits the cross-linking and excessive aggregation of polydopamine nanoparticles during Cu^2+^ coordination. As shown in Figure S5, energy dispersive X-ray spectroscopy (EDS) characterization of the Ti/PDT(Cu) sample further verified the homogeneous distribution of Cu elements throughout the coating. More importantly, this uniform and dense nanoscale microstructure provides a structural basis for the sustained and controlled release of Cu^2+^. Collectively, the SEM characterization results systematically demonstrate the successful stepwise fabrication of all polyphenol-based functional coatings on Ti substrates. The core findings are strictly limited to the observable morphological features: (1) TA incorporation can effectively regulate the polymerization and assembly behavior of polydopamine, significantly improving the density and uniformity of the coating nanoparticles and achieving nanoscale roughening of the coating; (2) Cu^2+^ loading in a single polydopamine matrix easily induces particle agglomeration and heterogeneous microstructure, while TA modification can effectively suppress this adverse effect and maintain the uniform nanostructure of the coating after Cu immobilization.

**Figure 4:**
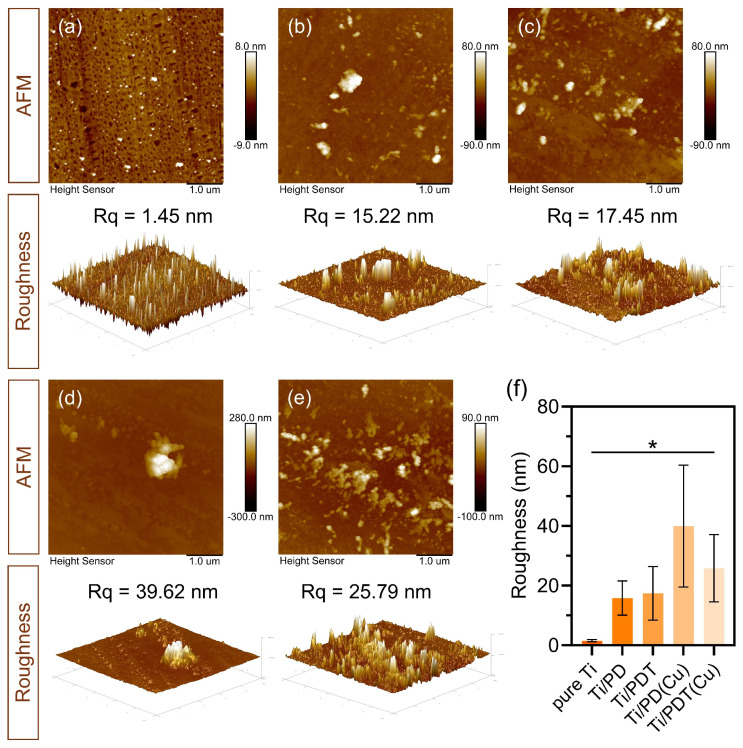
AFM characterization of pure Ti and functional coating-modified Ti substrates. Two-dimensional height images, corresponding 3D roughness topography, and root mean square roughness (Rq) values of (**a**) pure Ti, (**b**) Ti/PD, (**c**) Ti/PDT, (**d**) Ti/PD(Cu), and (**e**) Ti/PDT(Cu) samples (scan size: 5 μm × 5 μm). (**f**) Statistical results of surface roughness for all samples. All data are presented as mean ± standard deviation. * *p* < 0.05 indicates a significant difference between groups (*n* = 3 independent replicates).

AFM was performed to quantitatively characterize the three-dimensional (3D) surface topography and nanoscale roughness of the as-prepared samples, serving as a critical quantitative complement to the qualitative morphological observations from SEM. The 2D height images, corresponding 3D roughness topography, root mean square roughness (Rq) values, and statistical roughness results of all samples are presented in Figure 4. The mechanically polished pure Ti substrate (Figure 4a) exhibited an extremely flat and smooth surface with only faint nanoscale height fluctuations, as shown in both the 2D height map and 3D topography. The Rq value of pure Ti was as low as 1.45 nm, which established a baseline for evaluating the roughness changes induced by subsequent coating modifications and was fully consistent with the smooth surface with faint polishing scratches observed in our previous SEM characterization. After the deposition of the polydopamine coating (Ti/PD sample, Figure 4b), a significant increase in surface roughness was observed. The 2D height map showed uniform nanoscale protrusions distributed across the entire surface, and the Rq value increased markedly from 1.45 nm to 15.22 nm. The 3D topography revealed homogeneous low-amplitude height fluctuations without local sharp height changes, which corresponded to the dispersed polydopamine nanoparticles observed in SEM images. This result quantitatively confirmed the successful oxidative self-polymerization and stable deposition of dopamine on the Ti substrate. Then, incorporation of TA into the polydopamine matrix (Ti/PDT sample, Figure 4c) induced a further controllable increase in surface nanoscale roughness, which is the core finding aligned with the polyphenol chemistry-mediated roughening mechanism reported in previous studies. The Rq value of the Ti/PDT sample reached 17.45 nm, which was higher than that of the Ti/PD sample. The 2D height map showed denser and more uniformly distributed nanoscale protrusions, and the 3D topography exhibited a highly homogeneous height distribution without local excessive height fluctuations. This phenomenon can be attributed to the multiple hydrogen-bonding interactions between TA and polydopamine segments: as a natural polyphenol with abundant pyrogallol and catechol moieties, TA regulates the nucleation, growth, and assembly of polydopamine nanoparticles during dopamine polymerization, leading to a denser nanoparticle packing and a controllable increase in surface roughness. This quantitative roughness result is fully consistent with the denser and more uniform nanoparticle morphology observed in SEM images, and also complements the FTIR result that confirmed the successful introduction of TA and increased density of active hydroxyl groups on the coating surface. More importantly, this uniformly roughened nanostructure significantly increases the specific surface area of the coating, providing abundant and homogeneous active sites for the subsequent immobilization of Cu^2+^ via coordination interactions.

For the Cu-loaded Ti/PD(Cu) sample (Figure 4d), a dramatic and irregular increase in surface roughness was observed. The Rq value surged to 39.62 nm, which was more than twice that of the Ti/PD sample. The 2D height map showed an extremely heterogeneous surface topography with sharp local height mutations, with the height detection range reaching ±300 nm (far wider than that of other samples). The 3D topography revealed a large micron-scale agglomerate with a significant height difference from the surrounding coating, which directly corresponded to the irregular particle agglomeration observed in SEM images. This result quantitatively demonstrates that Cu^2+^ coordination in a single polydopamine matrix induces severe cross-linking and aggregation of adjacent polydopamine nanoparticles, leading to a highly heterogeneous surface structure. The limited number of active coordination sites in the pure polydopamine matrix cannot achieve uniform dispersion of Cu^2+^, resulting in local excessive cross-linking and uncontrolled roughness increase, which also verifies the successful coordination of Cu^2+^ with the polydopamine matrix as confirmed by FTIR characterization. Notably, the Ti/PDT(Cu) sample (Figure 4e) exhibited a significantly optimized and homogeneous surface topography after Cu^2+^ loading, effectively overcoming the structural heterogeneity problem of the Ti/PD(Cu) sample. The Rq value of Ti/PDT(Cu) was 25.79 nm, which was markedly lower than that of Ti/PD(Cu), and maintained a similar roughness level to the Ti/PDT sample. The 2D height map showed dense and uniform nanoscale protrusions without sharp local height mutations, with the height detection range returning to ±100 nm. The 3D topography exhibited a highly homogeneous height distribution, which was highly consistent with the topological characteristics of the Ti/PDT sample. This improvement can be attributed to the abundant phenolic hydroxyl groups introduced by TA, which provide a large number of additional and uniform coordination sites for Cu^2+^. The stable multi-component coordination structure formed among TA, polydopamine, and Cu^2+^ effectively inhibits the excessive cross-linking and aggregation of polydopamine nanoparticles during Cu^2+^ immobilization, thus maintaining the uniform nanoscale roughened structure of the coating while achieving stable Cu^2+^ loading. This result is fully consistent with the uniform nanoparticle morphology observed in SEM images. The statistical roughness results (Figure 4f) further quantitatively validated the above-mentioned trend of surface roughness evolution. The Ti/PD(Cu) sample exhibited a significantly higher surface roughness than other groups (* *p* < 0.05), while the Ti/PDT(Cu) sample maintained a well-regulated roughness level, confirming the critical role of TA in regulating the coating topological structure during Cu^2+^ loading. Collectively, the AFM characterization results provide quantitative and systematic evidence for the surface structure evolution of the functional coatings, with all interpretations strictly limited to the observable topological features and measurable roughness parameters. The core conclusions are as follows: (1) the successful deposition of polydopamine-based coatings was quantitatively verified by the significant increase in surface roughness compared with the pure Ti substrate; (2) TA incorporation realizes the controllable nanoscale roughening of the polydopamine coating, and constructs a uniformly roughened nanostructure with high specific surface area; (3) Cu^2+^ loading in a single polydopamine matrix induces severe particle aggregation and heterogeneous surface structure, while TA modification effectively suppresses this adverse effect and maintains the uniform topological structure of the coating after Cu^2+^ immobilization. These results, together with the previous FTIR and SEM characterizations, form a complete and mutually verified structural characterization system, and lay a quantitative structural foundation for the subsequent physicochemical property and biological performance evaluations of the functional coatings.

#### 3.1.3. Surface Properties and Stability Assays

**Figure 5:**
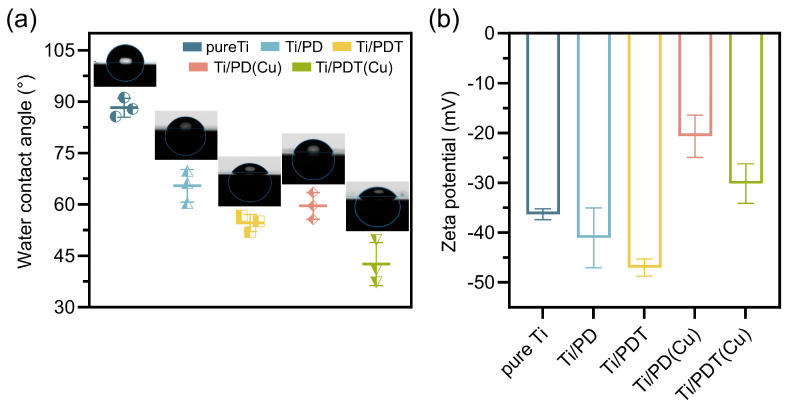
WCA and Zeta potential characterization of pure Ti and functional coating-modified Ti substrates. (**a**) Static WCA values for pure Ti, Ti/PD, Ti/PDT, Ti/PD(Cu), and Ti/PDT(Cu) samples; representative optical images of sessile water droplets on each sample surface are provided as insets. (**b**) Zeta potential measurements of different samples acquired in neutral aqueous solution. All data are presented as mean ± standard deviation (*n* = 3 independent replicates).

Surface wettability and interfacial charge are two dominant surface characteristics that govern the interfacial interactions between implant materials and the biological microenvironment, while the long-term structural stability of the coating is a prerequisite for reliable in vivo functional performance. Herein, we systematically evaluated the water contact angle, zeta potential, and physiological stability of the as-prepared polydopamine-based functional coatings on titanium substrates. Firstly, the surface wettability of different samples was characterized by static water contact angle measurements and the results are shown in Figure 5a. The pristine pure Ti substrate exhibited a hydrophobic surface with a WCA of 88.27 ± 2.78°, which was significantly reduced after the construction of polydopamine-based coatings. Specifically, the Ti/PD coating showed a decreased WCA of 65.47 ± 4.73°, attributed to the abundant hydrophilic phenolic hydroxyl and amino groups in the polydopamine matrix. The introduction of TA further improved surface hydrophilicity, with the WCA of the Ti/PDT coating reaching 54.60 ± 2.47°, as TA provides a higher density of catechol and hydroxyl groups that enhance surface hydration capacity. Notably, the WCA of the Ti/PD(Cu) was 59.6 ± 3.9° and the Ti/PDT(Cu) coating exhibited the best hydrophilicity among all groups, with the lowest WCA of 42.60 ± 6.27°. The excellent hydrophilicity of the Ti/PDT(Cu) coating is beneficial for optimizing interfacial biocompatibility, as hydrophilic surfaces can effectively reduce non-specific protein adsorption, platelet adhesion, and bacterial attachment, which are critical for alleviating thrombosis and infection, the two core complications of cardiovascular implants.

Subsequently, the surface charge properties of different samples in neutral aqueous solution were characterized by zeta potential measurements and the results are shown in Figure 5b. The pristine pure Ti substrate showed a negatively charged surface with a zeta potential of −36.33 ± 1.12 mV, originating from the deprotonation of hydroxyl groups on the surface titanium dioxide layer. After PDA coating, the Ti/PD sample exhibited an enhanced negative zeta potential of −41.03 ± 6.05 mV, due to the deprotonation of abundant phenolic hydroxyl groups in the PDA network. The incorporation of TA further increased the negative charge density, with the zeta potential of Ti/PDT reaching −47.03 ± 1.74 mV, as the large number of phenolic hydroxyl groups in TA provides more negatively charged sites after deprotonation under neutral conditions. In contrast, the Ti/PD(Cu) coating showed a significantly weakened negative zeta potential of −20.67 ± 4.16 mV, which is attributed to the chelation between positively charged Cu^2+^ and the catechol/amino groups in PDA, neutralizing part of the negative surface charge. For the Ti/PDT(Cu) coating, the zeta potential was maintained at −30.17 ± 3.94 mV, retaining a moderate negative charge compared with Ti/PD(Cu). This is because the introduction of TA provides sufficient catechol groups, which not only participate in Cu^2+^ chelation but also retain abundant deprotonated hydroxyl groups to maintain negative surface charge. It is well established that a moderately negatively charged surface can effectively inhibit the adhesion and activation of intrinsically negatively charged platelets via electrostatic repulsion, thus endowing the coating with improved antithrombotic potential.

Finally, the long-term structural stability of the functional coatings in a simulated physiological environment was evaluated by monitoring the thickness retention rate via spectroscopic ellipsometry after immersion in phosphate-buffered saline for 7 days and the results are shown in Figure S6. All polydopamine-based coatings maintained excellent structural integrity without obvious delamination or acute degradation during the 7-day immersion period. Specifically, the pure PDA coating (Ti/PD) showed a thickness retention rate of 75.62 ± 5.41% after 7 days. The Ti/PDT coating exhibited a significantly higher thickness retention rate of 91.18 ± 1.03%, which is ascribed to the dense cross-linking network formed between DA and TA via covalent bonding and hydrogen bonding, effectively suppressing the swelling and degradation of the PDA matrix. The Ti/PD(Cu) coating maintained a thickness retention rate of 82.74 ± 2.94% after 7 days, while the Ti/PDT(Cu) coating achieved the optimal structural stability with the highest thickness retention rate of 95.68 ± 0.40%. The superior stability of the Ti/PDT(Cu) coating is attributed to the robust dual cross-linking network: the covalent/hydrogen bonding between DA and TA, as well as the stable metal coordination chelation between catechol groups (from DA and TA) and Cu^2+^. This dense cross-linking structure not only enhances the mechanical integrity of the coating but also stabilizes the PDA aggregation structure to reduce its degradation and dissolution in the physiological environment, providing a reliable structural foundation for long-term in vivo implantation applications.

### 3.2. In Vitro Antibacterial Property

**Figure 6:**
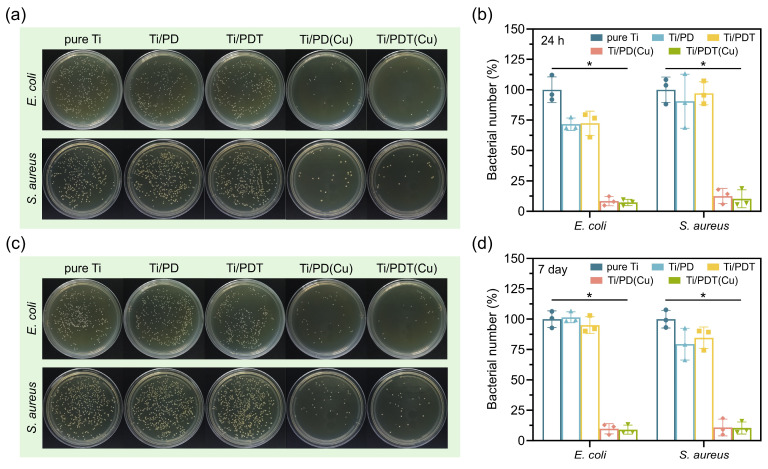
In vitro antibacterial performance of functionalized Ti substrates against *E. coli* and *S. aureus*. (**a**,**b**) Representative digital photographs of bacterial CFU on agar plates and corresponding quantitative analysis of relative bacterial number after 24 h co-incubation of as-prepared pure Ti, Ti/PD, Ti/PDT, Ti/PD(Cu), and Ti/PDT(Cu) samples with bacteria. (**c**,**d**) Representative CFU agar plate images and quantitative relative bacterial number for the same sample groups after 7 days of dynamic immersion in PBS, followed by a 24 h antibacterial co-incubation assay to evaluate the long-term antibacterial durability of the coatings. All data are presented as mean ± standard deviation. * *p* < 0.05 indicates a statistically significant difference between groups (*n* = 3 independent replicates).

Implant-associated infection remains one of the most life-threatening complications for long-term cardiovascular implants, particularly for LVADs, with a 5-year cumulative infection rate of up to 40.1% and infection-related mortality accounting for over 10% of all-cause death in LVAD recipients, as established in our preceding clinical context analysis [50]. The pathological core of such infections is bacterial adhesion and subsequent biofilm formation on the device surface, which is dominated by Gram-positive *S. aureus* and Gram-negative *E. coli*. To address this unmet clinical need, we systematically evaluated the bactericidal efficacy of the fabricated pure Ti, Ti/PD, Ti/PDT, Ti/PD(Cu), and Ti/PDT(Cu) substrates against both *E. coli* and *S. aureus* via the gold-standard spread plate colony counting assay. Both short-term (24 h direct co-incubation) antibacterial activity and long-term antibacterial stability (after 7 days of dynamic immersion PBS to simulate the in vivo physiological elution environment of long-term implantation) were characterized, with all results presented in Figure 6. For the short-term antibacterial assessment after 24 h of co-incubation with bacterial suspension, the representative optical images of bacterial colonies on agar plates (as shown in Figure 6a) provided intuitive visual evidence of the bactericidal effects across different sample groups. The pure Ti group exhibited dense and numerous bacterial colonies for both *E. coli* and *S. aureus*, confirming the negligible intrinsic antibacterial activity of the bare titanium substrate, which is consistent with the well-documented high infection susceptibility of unmodified titanium implants in clinical practice. Similarly, the non-copper-loaded groups (Ti/PD and Ti/PDT) showed only a slight reduction in colony formation, with no visually apparent bactericidal effect observed. This finding is aligned with existing literature reports: pristine PDA coatings only exhibit mild bacteriostatic rather than bactericidal activity, as they lack bactericidal active components and their weak antibacterial effect is only derived from limited adhesion interference to bacteria. Meanwhile, although TA has been reported to have mild antibacterial properties at high concentrations, the low content of TA incorporated in the Ti/PDT coating in this work is insufficient to exert a significant bactericidal effect. This design deliberately avoids the potential cytotoxicity of high-dose polyphenols, while leveraging TA to regulate the coating structure and copper ion coordination behavior as we originally designed.

**Figure 7:**
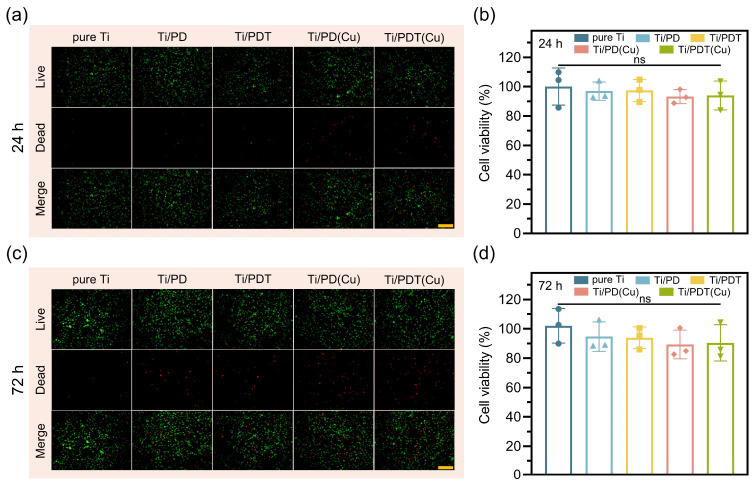
In vitro cytocompatibility assessment of functionalized Ti substrates. (**a**,**c**) Representative fluorescence micrographs of live/dead-stained L929 fibroblasts after 24 h and 72 h co-incubation with pure Ti, Ti/PD, Ti/PDT, Ti/PD(Cu), and Ti/PDT(Cu) samples. Viable cells are labeled with green fluorescence, and dead cells with red fluorescence. Scale bar: 200 μm. (**b**,**d**) Quantitative cell viability of L929 cells cultured with different samples for 24 h and 72 h, determined by CCK-8 assay. All data are expressed as mean ± standard deviation. ns denotes no statistically significant difference between groups (*n* = 3 biological replicates).

In sharp contrast, the copper immobilized groups (Ti/PD(Cu) and Ti/PDT(Cu)) displayed almost complete elimination of viable bacteria on the agar plates, with only sporadic colonies observed for both bacterial strains, demonstrating remarkable short-term bactericidal performance. Quantitative analysis of the relative bacterial number further validated the aforementioned qualitative results with statistical significance (as shown in Figure 7b). For *E. coli*, the relative bacterial viability of the Ti/PD and Ti/PDT groups was 71.63 ± 5.11% and 72.38 ± 9.99%, respectively, showing only mild bacterial inhibition without statistical significance compared with the pure Ti group. Whereas the Ti/PD(Cu) and Ti/PDT(Cu) groups achieved a dramatic reduction in bacterial viability, with the relative bacterial number decreased to only 8.34 ± 3.81% and 7.21 ± 2.55%, corresponding to a bacterial reduction rate of 91.66% and 92.79%, respectively, with statistically significant differences from the pure Ti group (* *p* < 0.05). For *S. aureus*, consistent antibacterial trends were observed: the Ti/PD and Ti/PDT groups showed negligible bacterial inhibition, with relative bacterial viability of 90.53 ± 22.23% and 97.08 ± 9.47%, respectively. In comparison, the Ti/PD(Cu) and Ti/PDT(Cu) groups exhibited outstanding bactericidal activity, with the relative bacterial number reduced to 12.59 ± 6.20% and 10.27 ± 7.35%, achieving a bacterial reduction rate of 87.41% and 89.73%, respectively (* p < 0.05).

Notably, the Ti/PDT(Cu) group showed slightly superior antibacterial efficacy compared with the Ti/PD(Cu) group for both bacterial strains, which can be directly attributed to the structural regulation effect of TA on the PDA coating, as reported in the previous work [49,51]. The introduction of TA accelerates dopamine polymerization, induces rapid roughening of the coating, increases the specific surface area, and provides more phenolic hydroxyl groups for copper ion chelation. This not only improves the loading capacity of copper ions, but also enhances the stability of copper ion coordination through the metal-phenolic network formed between TA and copper ions, thus enabling more efficient and sustained release of bactericidal active copper ions. The excellent broad-spectrum antibacterial activity of the copper-loaded coatings originates from the multi-target bactericidal mechanism of copper ions: copper ions can disrupt the integrity of bacterial cell membranes, leading to the leakage of intracellular contents; interfere with bacterial DNA replication and transcription to inhibit proliferation; block energy metabolism by inhibiting key enzymes in the bacterial respiratory chain; and induce reactive oxygen species (ROS) production to cause oxidative damage to bacterial biomacromolecules. Unlike single-target antibiotics, this multi-target bactericidal mechanism greatly reduces the risk of bacterial drug resistance, which is particularly critical for long-term implantable devices such as LVADs that require durable antibacterial protection. Furthermore, compared with the clinically widely used silver-based antibacterial coatings, our copper-based strategy avoids the inherent defects of silver coatings, including systemic cytotoxicity caused by silver ion accumulation, hemolysis, and platelet activation that increases thrombotic risk. More importantly, copper ions can simultaneously exert antibacterial activity and catalyze the release of NO for anticoagulation, realizing the integration of dual functions with a single active component.

For long-term implantable cardiovascular devices such as LVADs, long-term antibacterial stability is a core prerequisite for clinical translation, as most existing antibacterial coatings only exhibit short-term efficacy and fail to prevent late-onset implant infections that occur months to years after implantation. To verify the long-term antibacterial stability of the as-prepared coatings, we further evaluated the antibacterial performance of all samples after 7 days of dynamic immersion in PBS to simulate the in vivo physiological elution environment. As shown in the colony morphology images (Figure 6c), the pure Ti, Ti/PD, and Ti/PDT groups still exhibited dense and abundant bacterial colonies for both *E. coli* and *S. aureus* after 7 days of PBS immersion, indicating no sustained antibacterial activity. In contrast, the Ti/PD(Cu) and Ti/PDT(Cu) groups maintained excellent bactericidal performance, with only a tiny number of bacterial colonies observed on the agar plates, demonstrating robust long-term antibacterial stability of the copper-immobilized MPN coatings. Quantitative results (Figure 7d) further confirmed the sustained antibacterial efficacy of the copper-loaded coatings. For *E. coli*, after 7 days of PBS immersion, the relative bacterial viability of the Ti/PD and Ti/PDT groups was 101.57 ± 4.47% and 95.12 ± 6.93%, respectively, showing no bacterial inhibition effect compared with the pure Ti group. Meanwhile, the Ti/PD(Cu) and Ti/PDT(Cu) groups still maintained outstanding bactericidal activity, with the relative bacterial number of only 9.77 ± 4.21% and 9.07 ± 3.70%, corresponding to a bacterial reduction rate of 90.23% and 90.93%, respectively (* *p* < 0.05). For *S. aureus*, the Ti/PD and Ti/PDT groups showed only mild bacterial inhibition, with relative bacterial viability of 79.47 ± 13.08% and 84.65 ± 8.90%, respectively. At the same time, the Ti/PD(Cu) and Ti/PDT(Cu) groups retained excellent long-term bactericidal performance, with the relative bacterial number reduced to 10.90 ± 6.88% and 10.45 ± 4.95%, achieving a bacterial reduction rate of 89.10% and 89.55%, respectively (* *p* < 0.05). It is worth noting that there was no significant attenuation of antibacterial efficacy in both copper-loaded groups after 7 days of PBS immersion compared with the 24 h short-term results, which is a key advancement over traditional PDA-Cu coatings. As we discussed in the introduction, traditional PDA-Cu coatings suffer from critical drawbacks: single coordination mode between copper ions and PDA, uncontrollable copper ion release kinetics, initial burst release, and rapid loss of copper ions in physiological environments, leading to short-term cytotoxicity and rapid loss of antibacterial activity. In our design, the incorporation of TA constructs a more stable MPN structure with dual phenolic sources (PDA and TA), which provides more abundant coordination sites for copper ions, thus realizing sustained and controlled release of copper ions without obvious burst release or rapid loss. This stable release profile not only ensures long-term antibacterial efficacy but also avoids potential cytotoxicity caused by excessive copper ion burst release, laying a foundation for the excellent biocompatibility of the coating. From a clinical perspective, this long-term stable antibacterial performance has the potential to address the clinical challenge of late-onset LVAD-related infections, which account for a large proportion of infection-related adverse events in long-term LVAD recipients.

### 3.3. In Vitro Cytocompatibility

For long-term implantable cardiovascular devices such as artificial heart pumps, excellent cytocompatibility and non-cytotoxicity are the core prerequisites for clinical translation, as they directly determine the in vivo biosafety, endothelialization potential, and long-term service performance of the implants. In this study, the in vitro cytotoxicity of the as-fabricated functional coatings on Ti substrates was systematically evaluated using L929 mouse fibroblasts, in strict accordance with the ISO 10993-5 international standard for the biological evaluation of medical devices [52]. Qualitative Live/dead cell fluorescence staining and quantitative Cell Counting Kit-8 (CCK-8) assay were performed after 24 h and 72 h of co-culture, with all results presented in Figure 7. The quantitative cell viability results (Figure 7b,d) revealed that all polyphenol-based coating-modified Ti samples exhibited outstanding cytocompatibility without obvious cytotoxicity throughout the entire co-culture period. After 24 h of incubation, the relative cell viability of the pure Ti control group was normalized to 100.00 ± 12.67%, while the Ti/PD, Ti/PDT, Ti/PD(Cu), and Ti/PDT(Cu) groups maintained high relative cell viabilities of 96.93 ± 6.22%, 97.37 ± 7.56%, 93.20 ± 4.77%, and 93.97 ± 9.86%, respectively. With the extension of co-culture time to 72 h, the cell viability of all groups remained at a stable and favorable level: the pure Ti group reached 102.00 ± 11.81%, and the Ti/PD, Ti/PDT, Ti/PD(Cu), and Ti/PDT(Cu) groups still retained relative cell viabilities of 94.63 ± 10.11%, 93.93 ± 7.38%, 89.37 ± 9.80%, and 90.40 ± 12.34%, respectively. Notably, the relative cell viability of all experimental groups was far higher than the 80% cell viability threshold specified in the ISO 10993-5 standard for medical device cytocompatibility assessment, confirming the absence of cytotoxicity of the prepared coatings. Meanwhile, no statistically significant difference in cell viability was observed between all functional coating groups and the pure Ti control group (*p* > 0.05, marked as ns), further verifying that the as-prepared coatings did not induce adverse toxic effects on normal mammalian cells, even after prolonged co-culture.

The excellent cytocompatibility of the polyphenol-based coatings can be attributed to their rational molecular design and inherent bioactive chemical structure, which is in full alignment with our previous FTIR characterization results. For the Cu-free Ti/PD and Ti/PDT groups, the non-cytotoxic feature and favorable cell affinity originated from the abundant bioactive moieties on the coating surface, including phenolic hydroxyl groups and amino groups from the polydopamine matrix and TA [53]. As validated in our previous FTIR analysis, the successful oxidative self-polymerization of dopamine constructed a cross-linked PD network with inherent biocompatibility, while the introduction of TA via hydrogen bond-driven co-deposition did not damage the fundamental structure of PD. Instead, as reported in the previous study on rapid roughening of polydopamine nanocoatings via polyphenol chemistry, TA incorporation introduced a large number of additional pyrogallol and catechol groups and constructed a micro-nano roughened surface on the coating. These active groups and hierarchical surface structure provide sufficient adhesion sites for cell attachment and spreading, thus supporting the normal proliferation of L929 cells without inducing any toxic response, which explains the comparable cell viability between Ti/PD/Ti/PDT groups and the pure Ti control.

For the Cu-loaded Ti/PD(Cu) and Ti/PDT(Cu) groups, despite a slight decrease in cell viability compared with the Cu-free groups, they still maintained high cell viability far above the non-cytotoxic threshold, with no statistically significant difference from the pure Ti control. This result perfectly addresses the long-standing bottleneck of traditional antibacterial implant coatings: the irreconcilable trade-off between high antibacterial efficiency and adverse non-specific cytotoxicity to normal cells. As confirmed by our previous FTIR characterization and antibacterial test results, Cu^2+^ ions were immobilized in the polyphenol matrix via stable coordination bonds (Cu-O bonds) with deprotonated phenolic hydroxyl groups, rather than simple physical adsorption. This stable coordination structure enables a sustained and controlled release of Cu^2+^ ions, precisely maintaining the ion concentration within a safe therapeutic window: it can effectively kill pathogenic bacteria (as demonstrated in our antibacterial results) without causing toxic damage to normal mammalian cells. In particular, the Ti/PDT(Cu) group exhibited slightly higher cell viability and better stability than the Ti/PD(Cu) group, which can be ascribed to the abundant polyphenol moieties introduced by TA. These moieties provide more abundant and uniform coordination sites for Cu^2+^ ions, leading to more stable Cu immobilization, avoiding local burst release of Cu ions, and further optimizing the cytocompatibility of the antibacterial coating.

The qualitative live/dead cell fluorescence staining results (Figure 7a,c) provided direct visual evidence for the excellent cytocompatibility of the as-fabricated coatings, which was in full agreement with the quantitative CCK-8 results. After 24 h and 72 h of co-culture, all sample groups were dominated by cells with bright green fluorescence (live cells with intact cell membranes), while almost no red fluorescent dead cells with damaged membranes were observed, indicating an extremely low cell death rate in all groups. Moreover, the cells on all functional coating surfaces showed uniform distribution and normal fibroblast morphology, with a cell density comparable to that of the pure Ti control group. Notably, the cell density of all groups at 72 h was significantly higher than that at 24 h, demonstrating that L929 cells could adhere, spread, and proliferate normally on the surface of all functional coatings, further confirming the non-cytotoxic nature and favorable cell affinity of the as-prepared coatings.

**Figure 8:**
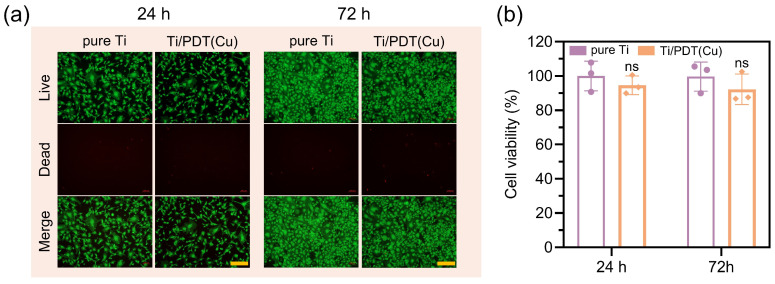
In vitro cytocompatibility assessment of functionalized Ti substrates. (**a**) Representative fluorescence micrographs of live/dead stained HUVECs after 24 h and 72 h co-incubation with pure Ti and Ti/PDT(Cu) samples. Viable cells are labeled with green fluorescence, and dead cells with red fluorescence. Scale bar: 200 μm. (**b**) Quantitative cell viability of HUVECs cultured with different samples for 24 h and 72 h, determined by CCK-8 assay. All data are expressed as mean ± standard deviation. ns denotes no statistically significant difference between groups (*n* = 3 biological replicates).

To further evaluate the in vitro biosafety of the functional coatings for cardiovascular applications, human umbilical vein endothelial cells (HUVECs), the primary cell type that directly interacts with cardiovascular implant surfaces, were employed to conduct additional cytotoxicity assays (Figure 8). The qualitative live/dead fluorescence staining results (Figure 8a) were highly consistent with the findings from L929 fibroblasts. After 24 h and 72 h of co-culture, all sample groups were predominantly populated by cells emitting bright green fluorescence, indicating intact cell membranes and viable status, while almost no red fluorescent dead cells with compromised membranes were detected in any group. Furthermore, HUVECs cultured on both pure Ti and Ti/PDT(Cu) surfaces exhibited typical cobblestone-like endothelial cell morphology, uniform distribution, and comparable cell density. Notably, the cell density in all groups increased significantly from 24 h to 72 h, demonstrating that HUVECs could adhere, spread, and proliferate normally on the Ti/PDT(Cu) coating surface. The quantitative CCK-8 assay results (Figure 8b) further corroborated the excellent cytocompatibility of the Ti/PDT(Cu) coating towards endothelial cells. At 24 h, the cell viability of the Ti/PDT(Cu) group was 94.63 ± 5.46%, which showed no statistically significant difference (ns) compared with the pure Ti control group (100.00 ± 8.65%). After 72 h of incubation, the cell viability of the Ti/PDT(Cu) group remained at 92.23 ± 8.90%, still comparable to that of the pure Ti group (100 ± 8.51%) with no significant statistical difference. Importantly, the cell viability values of all groups were well above the 70% threshold specified in the ISO 10993-5 international standard for medical devices, confirming the absence of obvious cytotoxicity of the Ti/PDT(Cu) coating against endothelial cells. Collectively, the HUVEC cytotoxicity results, together with the previous L929 fibroblast data, comprehensively demonstrated that the optimized Ti/PDT(Cu) coating possesses excellent and broad-spectrum cytocompatibility towards both fibroblasts and vascular endothelial cells. Given the critical role of endothelial cells in preventing thrombosis and promoting rapid endothelialization of cardiovascular implants, the favorable compatibility of the Ti/PDT(Cu) coating with HUVECs is of particular significance. These findings further strengthen the biosafety foundation for the subsequent blood compatibility evaluation and in vivo preclinical studies, and more convincingly validate the great application potential of this polyphenol-based antibacterial coating in artificial heart pumps and other cardiovascular implant devices.

### 3.4. Hemocompatibility

For long-term blood-contacting implantable devices such as LVADs and vascular stents, exceptional hemocompatibility is a fundamental prerequisite to avoid post-implantation thrombotic complications and ensure the long-term functional stability of the device. When a foreign material comes into contact with blood, it immediately triggers non-specific adsorption of plasma proteins, which in turn induces platelet adhesion, activation and aggregation, and may cause damage and rupture of red blood cells. These events ultimately initiate the intrinsic coagulation cascade, leading to thrombus formation, device failure, and even life-threatening thromboembolic events in patients. Nitric oxide, a key signaling molecule endogenously secreted by healthy vascular endothelial cells, exerts a potent inhibitory effect on platelet adhesion and activation by upregulating the cyclic guanosine monophosphate (cGMP) signaling pathway in platelets. Meanwhile, it also possesses multiple vascular protective functions, including vasodilation and inhibition of smooth muscle cell overgrowth, making it an ideal bioactive moiety for the construction of antithrombotic material surfaces. Herein, we systematically evaluated the hemocompatibility of different functionalized titanium substrates through hemolysis assay and platelet adhesion test, and combined with the catalytic NO generation performance (as shown in Figure 8), comprehensively verified the antithrombotic property and blood biosafety of the Ti/PDT(Cu) coating.

#### 3.4.1. Hemolysis Assay

The hemolysis ratio is a core indicator to evaluate the degree of damage to red blood cells (RBCs) caused by implant materials, and also the primary criterion for judging the blood biosafety of materials in the ISO 10993-4 standard, which clearly stipulates that materials with a hemolysis ratio of less than 5% meet the blood compatibility requirements for clinical blood-contacting applications. As shown in Figure 8b, the deionized water positive control group exhibited a 100% hemolysis ratio, while the saline negative control group showed no obvious hemolysis phenomenon. The hemolysis ratios of all functionalized titanium substrates were far below the 5% safety threshold. Specifically, the hemolysis ratios of the pure Ti, Ti/PD and Ti/PDT groups were only 0.30 ± 0.02%, 0.42 ± 0.09% and 0.37 ± 0.10% (Figure 8b), respectively, indicating that the polydopamine/tannic acid base layer itself does not cause significant mechanical or chemical damage to RBCs, and possesses intrinsic excellent blood biosafety. After the introduction of copper ion catalytic sites, the hemolysis ratios of the Ti/PD(Cu) and Ti/PDT(Cu) groups were 1.85 ± 0.55% and 0.97 ± 0.22% (Figure 8b), respectively, which were still significantly lower than the ISO safety standard, consistent with the hemolysis performance of previously reported copper-based NO catalytic coatings. Notably, the hemolysis ratio of the Ti/PDT(Cu) group was significantly lower than that of the Ti/PD(Cu) group. This can be attributed to the stable metal-phenolic network formed between TA and copper ions, which effectively limits the excessive burst release of copper ions and reduces the potential oxidative damage of free copper ions to RBCs [54,55]. Meanwhile, the inherent antioxidant activity of TA itself further alleviates RBC rupture mediated by oxidative stress. The above results fully demonstrate that the prepared Ti/PDT(Cu) functional coating has excellent hemocompatibility and meets the blood contact requirements of long-term implantable medical devices.

To further elucidate the advancements and originality of the developed Ti/PDT(Cu) coating, Table 1 presents a comprehensive comparison with recent state-of-the-art metal-phenolic and PDA-Cu coatings (2022–2025) [33,54,55]. While copper-mediated antibacterial and NO-generating networks have been widely reported, most conventional systems suffer from slow deposition kinetics (typically 24–48 h) and are prone to initial ion burst release, which compromises long-term physiological stability and hemocompatibility. By synergistically integrating TA into the polymerization process, our one-step strategy significantly reduces the deposition time to 8 h. Furthermore, the robust dual-crosslinked structure effectively traps Cu^2+^ within the metal-phenolic network, preventing acute ion leakage and enabling an exceptional balance of sustained antibacterial efficacy (>89% retention after 7 days), durable NO generation, and ultralow hemolysis (0.97%).

#### 3.4.2. Platelet Adhesion and Antithrombogenic Performance

Platelet adhesion, activation and aggregation are the initiating and core steps of thrombus formation. Upon blood contact with the material surface, plasma fibrinogen adsorbed on the surface binds to platelets through membrane receptors, inducing the transformation of platelets from a resting discoid shape to an activated spreading state. The activated platelets release granule contents and trigger the aggregation cascade, ultimately leading to thrombus formation. Therefore, inhibiting platelet adhesion and activation is a key indicator to evaluate the antithrombotic performance of biomaterials. Our results showed that the copper ion-functionalized coatings can continuously catalyze the decomposition of endogenous S-nitrosothiols (RSNOs) to generate NO under physiological conditions. As shown in Figure 8a, within 14 days, the cumulative NO release of the Ti/PDT(Cu) group reached 43.85 ± 2.36 μM, corresponding to an average NO flux of 1.6 × 10^−10^ mol/cm^2^·min. This value falls well within the physiological NO release range of healthy vascular endothelial cells (0.5–4 × 10^−10^ mol/cm^2^·min) cited in the introduction, while the control groups without copper ions only showed negligible NO generation (Figure 8a). This sustained and stable NO release behavior accurately mimics the NO secretion function of healthy vascular endothelium and effectively inhibits platelet activation and adhesion through the cGMP signaling pathway. A limitation of this study is the lack of direct copper release measurements. However, the sustained antibacterial efficacy, stable NO generation, excellent cytocompatibility and low hemolysis observed over 7 days collectively suggest a controlled release of copper ions from the PDA/TA network, maintaining concentrations within the safe and effective therapeutic window. This is consistent with previous reports on metal-phenolic network coatings, where metal ions are released slowly through the gradual degradation of the organic matrix. Quantitative copper release analysis will be performed in our future work.

**Figure 9:**
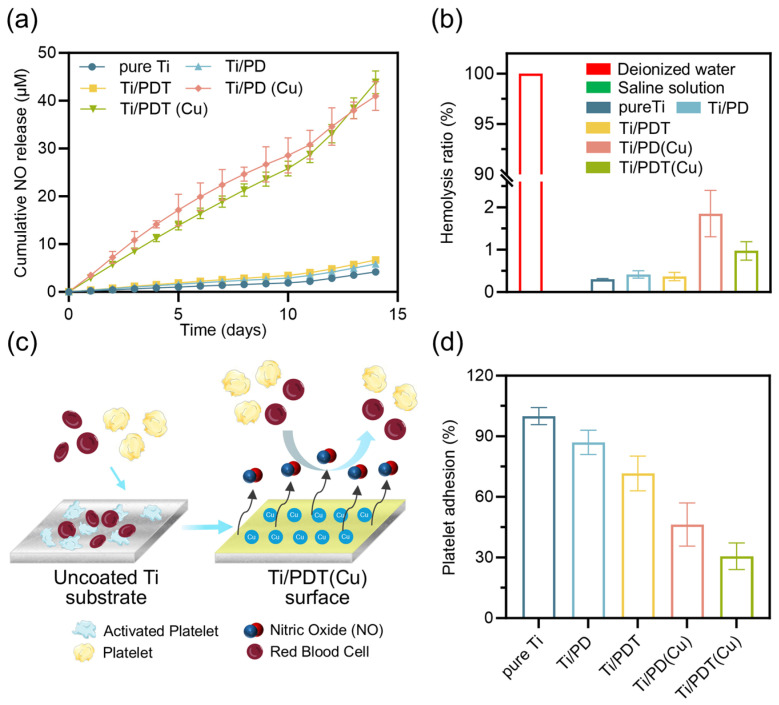
Hemocompatibility evaluation of the functionalized Ti substrates. (**a**) 14-day cumulative NO release profiles of different samples. (**b**) Hemolysis ratios of all sample groups. (**c**) Schematic diagram of the anti-platelet adhesion mechanism of the Ti/PDT(Cu) coating. (**d**) Relative platelet adhesion rates on different sample surfaces. All data are expressed as mean ± standard deviation. ns denotes no statistically significant difference between groups (*n* = 3 biological replicates).

The quantitative results of the platelet adhesion assay are shown in Figure 9d. Taking the platelet adhesion rate of the pure Ti group as 100%, the platelet adhesion rates of the Ti/PD and Ti/PDT groups decreased to 87.00 ± 5.96% and 71.63 ± 8.62%, respectively. This is attributed to the hydrophilic modification of polydopamine and tannic acid, which reduces the non-specific adsorption of plasma proteins to a certain extent, thereby lowering platelet adhesion. After the introduction of copper ion catalytic sites, the platelet adhesion rates of the Ti/PD(Cu) and Ti/PDT(Cu) groups showed an extremely significant reduction, to only 46.33 ± 10.68% and 30.60 ± 6.60%, respectively, and both groups showed a statistically significant difference compared with the pure Ti control group (* *p* < 0.05).

Among them, the Ti/PDT(Cu) coating exhibited the optimal anti-platelet adhesion performance, with a platelet adhesion inhibition rate of up to 69.4%, which was significantly better than that of the Ti/PD(Cu) group. This excellent performance originates from multiple synergistic mechanisms [58,59]: (1) The TA-copper ion MPN enables stable and continuous catalytic NO generation, which strongly inhibits platelet activation through an endothelium-mimicking signaling pathway; (2) The polyphenol structure of TA and the adhesive property of polydopamine synergistically construct a hydrophilic interface, reducing the surface adsorption of procoagulant proteins such as fibrinogen; (3) TA itself possesses a certain anti-platelet aggregation activity, which further enhances the antithrombotic effect of the coating. The mechanism of the interaction between platelets and the material surface is illustrated in Figure 9c. On the uncoated pure Ti substrate, platelet adhesion and activation were readily triggered, and a large number of platelets spread on the material surface and presented a highly activated dendritic morphology, which initiated the thrombus formation cascade. In contrast, the Ti/PDT(Cu) surface effectively inhibited platelet adhesion and activation through continuous catalytic NO release, maintaining platelets in a resting round morphology, and significantly reducing the adhesion and aggregation of activated platelets on the material surface, thus blocking the initial link of thrombus formation from the source. In summary, the Ti/PDT(Cu) functional coating achieves continuous NO generation under physiological conditions through the stable catalytic effect of the TA-copper ion MPN. Combined with the hydrophilic modification and bioactivity of polyphenol materials, it not only meets the hemocompatibility requirements of the ISO 10993-4 standard, but also exhibits excellent anti-platelet adhesion and antithrombotic properties. This work provides an efficient and safe surface modification strategy to solve the post-implantation thrombotic complications of long-term blood-contacting implant devices such as LVADs.

To further highlight the advancements of the developed Ti/PDT(Cu) coating, a direct comparison with traditional PDA- or TA-based titanium modifications reveals several distinct advantages. First, regarding fabrication time, the incorporation of TA into our system accelerates the deposition process to merely 8 h, overcoming the inherently slow polymerization kinetics of conventional PDA coatings which typically require >24 h. Second, the robust dual-crosslinked network significantly enhances long-term stability; while pure PDA coatings are prone to degradation in physiological environments (exhibiting only a 75.62% thickness retention after 7 days), the Ti/PDT(Cu) coating maintains an outstanding 95.68% structural integrity. Third, in terms of antibacterial efficacy, conventional PDA-Cu coatings often suffer from uncontrolled initial burst release and rapid loss of functionality. Conversely, the stable metal-phenolic coordination in our coating enables sustained bactericidal activity, retaining >89% efficacy even after 7 days of continuous physiological elution. Finally, this highly controlled release profile translates to superior hemocompatibility. The Ti/PDT(Cu) modification achieves a 69.4% inhibition of platelet adhesion and an ultralow hemolysis ratio of 0.97% (significantly lower than the 1.85% of standard Ti/PD(Cu) coatings). This has the potential to address the critical trade-off between potent long-term antibacterial capability and stringent hemocompatibility required for blood-contacting cardiovascular device applications.

## 4. Conclusions and Outlook

In summary, targeting the critical complications of thromboembolism and device-related infection in long-term left ventricular assist devices (LVADs), we developed a facile, one-step rapid co-polymerization strategy based on mussel-inspired polyphenol chemistry to construct a copper-integrated polydopamine/tannic acid (Ti/PDT(Cu)) nanocomposite coating. By incorporating tannic acid, we synergistically accelerated dopamine oxidative polymerization and drastically shortened the fabrication period. This approach constructs a robust dual-crosslinked network through covalent/hydrogen bonds and metal-phenolic coordination, endowing the coating with a uniform nanoscale roughened structure, excellent hydrophilicity, and superior long-term structural integrity in physiological environments. Crucially, the optimized coating design overcomes the inherent drawbacks of conventional PDA-Cu systems—such as uncontrollable ion burst release and poor physiological stability. It provides durable, broad-spectrum bactericidal efficacy while enabling sustained, endothelium-mimicking catalytic nitric oxide generation. This results in an exceptional balance of potent anti-platelet adhesion, ultralow hemolysis, and outstanding cytocompatibility. Ultimately, this work realizes the synergistic integration of antithrombotic and antibacterial dual functions specifically tailored to the demanding conditions of high shear stress and prolonged implantation. Beyond LVADs, this facile and scalable metal-phenolic network design emphasizes a broader significance: it offers a robust surface modification platform capable of addressing complex device-blood interface challenges across a wide spectrum of cardiovascular implants. Future research will focus on in vivo validation under high shear flow conditions, optimizing NO catalytic kinetics, and exploring the scalable modification of complex-shaped implant architectures to further propel the clinical translation of this multifunctional coating system.

## Figures and Tables

**Table 1:** Quantitative comparison of recent PDA/MPN-based copper functionalized coatings (2022–2025) [33,56,57] with the proposed Ti/PDT(Cu) system.

Coating System	Deposition Time	Antibacterial Efficiency	NO Generation Capacity (μM)	Hemolysis Ratio
TiDA/Cu-Hep@Ag	29 h	98.14%	65	1.42%
ABP@Cu-NE/HD	over 48 h	98%	60	/
PET/TA/Cu(II)	over 36 h	97%	25	0.33%
Ti/PDT(Cu)	8 h	92.79%	45	0.97%

## Data Availability

The original contributions presented in this study are included in the article/Supplementary Material. Further inquiries can be directed to the corresponding author.

## Supplementary Materials

The following supporting information can be downloaded at: https://www.mdpi.com/article/10.3390/biom16070953/s1, Figure S1. UV-vis spectroscopic monitoring of dopamine polymerization kinetics. Time-dependent absorbance at 420 nm, corresponding to the formation of conjugated indole structures during dopamine oxidative polymerization, for four reaction systems: pure dopamine (DA), dopamine with tannic acid (DA + TA), dopamine with copper ions (DA + Cu), and dopamine with both tannic acid and copper ions (DA + TA + Cu). Figure S2. Thickness of polydopamine-based coatings on titanium substrates after 8 h of deposition. The thickness values of Ti/PD, Ti/PDT, Ti/PD(Cu), and Ti/PDT(Cu) coatings, measured by spectroscopic ellipsometry. Data are presented as mean ± standard deviation (*n* = 3). * *p* < 0.05 indicates a significant difference between groups. Figure S3. High-resolution Cu 2p spectrum of the Ti/PD(Cu) coating. Figure S4. EPR spectra of the Ti/PDT and Ti/PDT(Cu) sample. Figure S5. EDS mapping of Cu for the Ti/PDT(Cu) sample. Figure S6. Long-term structural stability of polydopamine-based coatings under simulated physiological conditions. Thickness retention rate of Ti/PD, Ti/PDT, Ti/PD(Cu), and Ti/PDT(Cu) coatings as a function of immersion time (1–7 days) in phosphate-buffered saline (PBS, pH 7.4) at 37 °C. The initial thickness of each coating on day 1 was normalized to 100%, and subsequent thickness values were calculated relative to this baseline. Data are presented as mean ± standard deviation (*n* = 3 independent replicates).
