# Supplementary material for: Copper Integrated PDA-TA Nanocoating via One-Step Rapid Polymerization on Titanium for Anti-Thrombotic and Antibacterial Properties
Source: Biomolecules. 2026 Jun 27;16(7):953. doi: 10.3390/biom16070953 (PMC13407066; doi:10.3390/biom16070953)
Supplement: Supplementary file 1 [file biomolecules-16-00953-s001.zip › biomolecules-4353799-supplementary.pdf]

Supporting Information for:

Copper Integrated PDA-TA Nanocoating via One-Step Rapid Polymerization on Titanium for Anti-Thrombotic and Antibacterial Properties

Chuangxin Huang,[a,b] Xin Liu,[a,*] Zerong Zhang,[a] Yanjun Liu,[a,b] Qi Chen,[a] Jianli Meng,[a] Qiuliang Wang,[a,b,c]

a Ganjiang Innovation Academy, Chinese Academy of Sciences, Ganzhou 341119, China.

b School of Rare Earths, University of Science and Technology of China, Hefei 230026, China.

c Institute of Electrical Engineering, Chinese Academy of Sciences, Beijing 100190, China.

* Corresponding author.

E-mail addresses: xliu@gia.cas.cn (X. Liu)

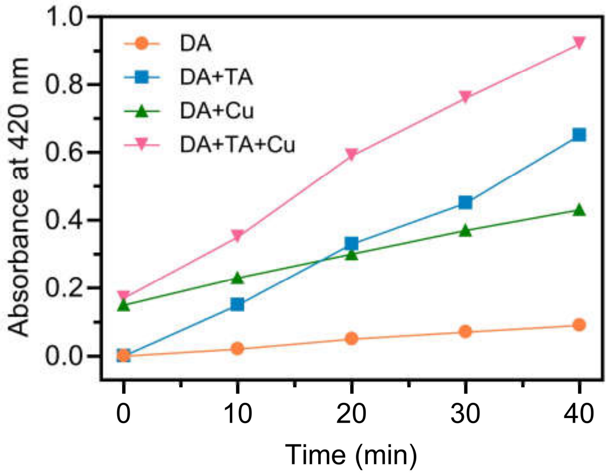

Figure S1. UV-vis spectroscopic monitoring of dopamine polymerization kinetics. Time-dependent absorbance at 420 nm, corresponding to the formation of conjugated indole structures during dopamine oxidative polymerization, for four reaction systems: pure dopamine (DA), dopamine with tannic acid (DA+TA), dopamine with copper ions (DA+Cu), and dopamine with both tannic acid and copper ions (DA+TA+Cu).

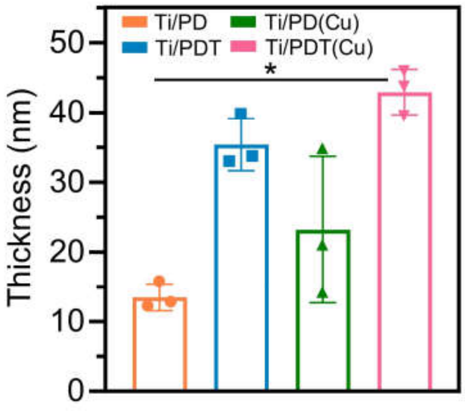

Figure S2. Thickness of polydopamine-based coatings on titanium substrates after 8 h of deposition. The thickness values of Ti/PD, Ti/PDT, Ti/PD(Cu), and Ti/PDT(Cu) coatings, measured by spectroscopic ellipsometry. Data are presented as mean ± standard deviation (n = 3). *$p < 0.05$ indicates a significant difference between groups.

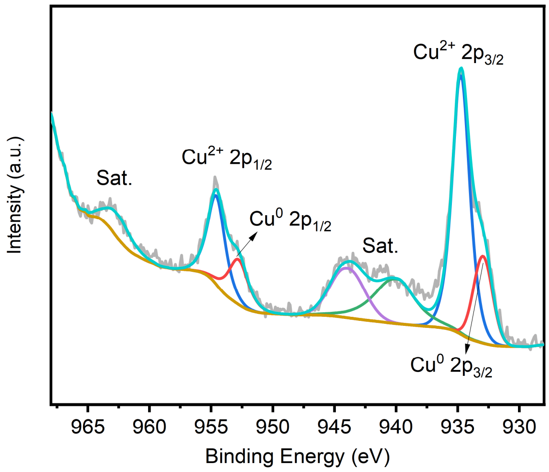

Figure S3. High-resolution Cu 2p spectrum of the Ti/PD(Cu) coating.

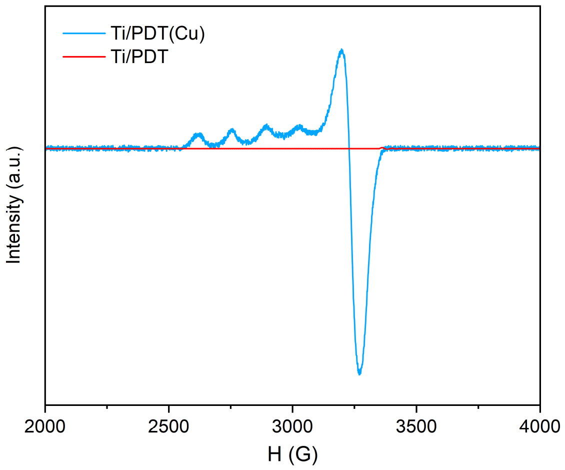

Figure S4. EPR spectra of the Ti/PDT and Ti/PDT(Cu) sample.

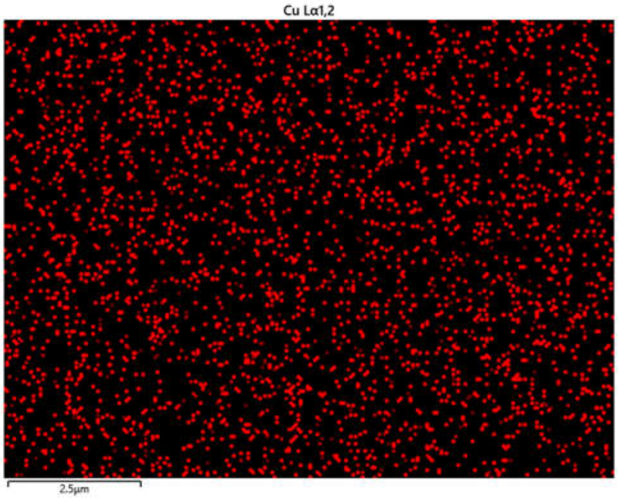

Figure S5. EDS mapping of Cu for the Ti/PDT(Cu) sample.

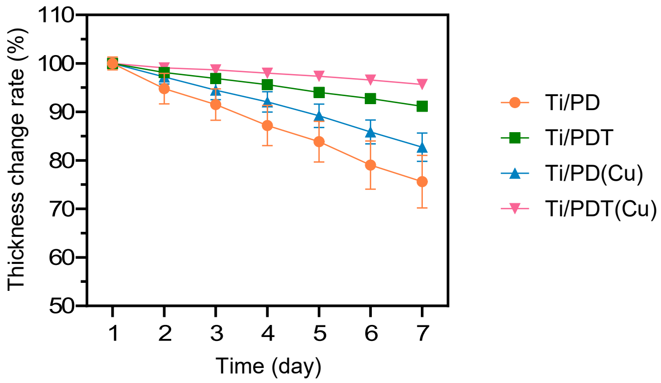

Figure S6. Long-term structural stability of polydopamine-based coatings under simulated physiological conditions. Thickness retention rate of Ti/PD, Ti/PDT, Ti/PD(Cu), and Ti/PDT(Cu) coatings as a function of immersion time (1-7 days) in phosphate-buffered saline (PBS, pH 7.4) at 37 °C. The initial thickness of each coating on day 1 was normalized to 100%, and subsequent thickness values were calculated relative to this baseline. Data are presented as mean ± standard deviation (n = 3 independent replicates).
